# Canine Babesiosis Caused by Large *Babesia* Species: Global Prevalence and Risk Factors—A Review

**DOI:** 10.3390/ani13162612

**Published:** 2023-08-13

**Authors:** Wojciech Zygner, Olga Gójska-Zygner, Justyna Bartosik, Paweł Górski, Justyna Karabowicz, Grzegorz Kotomski, Luke J. Norbury

**Affiliations:** 1 Division of Parasitology and Parasitic Diseases, Department of Preclinical Sciences, Institute of Veterinary Medicine, Warsaw University of Life Sciences—SGGW, Ciszewskiego 8, 02-786 Warsaw, Poland; justyna_bartosik@sggw.edu.pl (J.B.); pawel_gorski@sggw.edu.pl (P.G.); justyna_karabowicz@sggw.edu.pl (J.K.); 2 Labros Veterinary Clinic, Św. Bonifacego 92, 02-940 Warsaw, Poland; olgazygner@yahoo.pl (O.G.-Z.); gkotomski@o2.pl (G.K.); 3 Department of Biosciences and Food Technology, School of Science, STEM College, RMIT University, Bundoora, VIC 3083, Australia; luke.norbury2@rmit.edu.au

**Keywords:** canine babesiosis, large *Babesia*, *B. canis*, *B. vogeli*, *B. rossi*, *B. coco*, prevalence, risk factors

## Abstract

**Simple Summary:**

Four species of large *Babesia* cause canine babesiosis (*B. canis*, *B. rossi*, *B. vogeli*, and the informally named *B. coco*). Although canine babesiosis has a worldwide distribution, different species occur in specific regions: *B. rossi* in sub-Saharan Africa, *B. canis* in Europe and Asia, and *B. coco* in the Eastern Atlantic United States, while *B. vogeli* occurs in Africa, southern parts of Europe and Asia, northern Australia, southern regions of North America, and in South America. *B. vogeli* is the most prevalent large *Babesia* species globally. The most important risk factors for infection by large *Babesia* spp. include living in rural areas, kennels or animal shelters, or regions endemic for the infection, the season of the year (which is associated with increased tick activity), infestation with ticks, and lack of treatment with acaricides.

**Abstract:**

Canine babesiosis is a disease caused by protozoan pathogens belonging to the genus *Babesia*. Four species of large *Babesia* cause canine babesiosis (*B. canis*, *B. rossi*, *B. vogeli*, and the informally named *B. coco*). Although canine babesiosis has a worldwide distribution, different species occur in specific regions: *B. rossi* in sub-Saharan Africa, *B. canis* in Europe and Asia, and *B. coco* in the Eastern Atlantic United States, while *B. vogeli* occurs in Africa, southern parts of Europe and Asia, northern Australia, southern regions of North America, and in South America. *B. vogeli* is the most prevalent large *Babesia* species globally. This results from its wide range of monotropic vector species, the mild or subclinical nature of infections, and likely the longest evolutionary association with dogs. The most important risk factors for infection by large *Babesia* spp. include living in rural areas, kennels or animal shelters, or regions endemic for the infection, the season of the year (which is associated with increased tick activity), infestation with ticks, and lack of treatment with acaricides.

## 1. Introduction

Canine babesiosis is a tick-borne disease caused by the protozoan intraerythrocytic parasites of the genus *Babesia*. During the pathogen’s life cycle, ticks are the final hosts (zygote formation occurs in a tick’s intestine) and dogs are intermediate hosts (asexual division of the parasite occurs inside red blood cells). There are eight *Babesia* species that cause infection in dogs: *Babesia canis* Piana and Galli-Valerio, 1895; *Babesia vogeli* Reichenow, 1937; *Babesia rossi* Nuttall, 1910; *Babesia coco* (unofficial name) Birkenheuer, Neel, Ruslander, Levy, and Breitschwerdt, 2004; *Babesia negevi* Baneth, Nachum-Biala, Birkenheuer, Schreeg, Prince, Florin-Christensen, Schnittger, and Aroch, 2020; *Babesia gibsoni* Patton, 1910; *Babesia conradae* Kjemtrup, Wainwright, Miller, Penzhorn, and Carreno, 2006; and *Babesia vulpes* Baneth, Florin-Christensen, Cardoso, and Schnittger, 2015 [1,2]. *Babesia* species are grouped by morphology as either large or small. The first four *Babesia* species listed above are considered large piroplasms owing to the fact that their developmental stages, such as trophozoites and merozoites, are bigger than these stages in the second group of *Babesia*, i.e., the small *Babesia* species, which includes *B. gibsoni*, *B. conradae*, and *B. vulpes* [3]. The developmental stages of *B. negevi*, recently described by Baneth et al. [2], are smaller than large babesiae but larger than the small *Babesia* species.

Although *B. coco* is an unofficial name for an unnamed babesia parasite, some authors use it as a formal species name [4,5]. In some studies, other *Babesia* species have been found to have infected dogs. For instance, DNA from equine *Babesia caballi* Nuttall and Strickland, 1910, or the badger-infecting “*Babesia* sp. Meles-Hu1”, have been detected in the blood of dogs [6,7].

The canine large *Babesia* species cannot be differentiated using light microscopy via blood smear examination, and historically these piroplasms were considered one species, *B. canis*, which was divided into subspecies such as *B. canis canis*, *B. canis vogeli*, and *B. canis rossi*. Nowadays, these subspecies are considered separate species, and molecular techniques are used both in pathogen recognition and the determination of infection prevalence [1].

Depending on the species of the parasite and the immune status of the host, infection may lead to mild, moderate, or severe disease. *B. rossi* causes the most severe form of the disease in domestic dogs. Alternatively, *B. vogeli* infection may be mild or even subclinical in adult animals, but in young dogs may cause severe anemia. Infections caused by *B. canis* are typically milder than those caused by *B. rossi*; however, both pathogens can cause acute babesiosis [3]. The pathogenicity of *Babesia* sp. (Coco) is unknown, due to the low number of identified cases. However, disease caused by this species has been recognized only in splenectomized dogs or dogs after chemotherapy [8,9,10,11]. Thus, it seems probable that infection leads to the development of the disease only in immunosuppressed dogs [3].

Severe babesiosis, like human malaria and sepsis, is considered an immune-mediated disease which is characterized by cytokine storms and tissue hypoxia in various organs [12,13,14,15]. The disease may lead to anemia, coagulopathy, kidney injury, hepatopathy, pancreatitis, cardiac disorders, pulmonary edema, cerebral complications, endocrine disorders, systemic inflammatory response syndrome, multiple organ dysfunction syndrome, and septic shock in infected dogs [12,16,17,18,19,20,21,22,23,24,25,26,27,28]. Infections caused by large *Babesia* species have been recognized in dogs on all continents, except Antarctica [1].

The purpose of this review was to present the occurrence and diversity of the canine large *Babesia* spp. in the world, to show the gaps in knowledge regarding the occurrence of these parasites in the world, and to show which species is the most prevalent in the world, and why.

## 2. Prevalence of *Babesia rossi*

The pathogen is transmitted by the tick *Haemaphysalis elliptica* Koch, 1844, and probably *Haemaphysalis leachi* Audouin, 1826. In Nigeria, *B. rossi* DNA has also been detected in *Rhipicephalus sanguineus* Latreille, 1806, which is the most prevalent canine tick species in that country [1,29,30,31]. While Kamani et al. [31] initially considered this tick as a possible vector of *B. rossi* in Nigeria, this view was altered during further studies which also showed transovarial transmission of *B. rossi* in *H. leachi* but not in *R. sanguineus* [30]. In Turkey, *B. rossi* DNA has also been detected in ticks *Haemaphysalis parva* Neumann, 1897 [32,33].

Although *B. rossi* infections were initially noted in side-striped jackals (*Canis adustus* Sundevall, 1847), the black-backed jackal (*Canis mesomelas* Schreber, 1775) is considered the natural host of *B. rossi* [34,35]. However, Viljoen et al. [36] did not detect *B. rossi* DNA in blood samples collected post-mortem from 43 free-ranging black-backed jackals in South Africa. The African wild dog (*Lycaon pictus* Temminck, 1820) is another host of *B. rossi* in South Africa [37,38]. However, according to Shabangu et al. [38], African wild dogs are not a significant reservoir of infection, and a separate study from various sub-Saharan African countries (Botswana, Namibia, South Africa, and Kenya) did not identify *Babesia* infections among 154 presumably asymptomatic African wild dogs [39]. Ciuca et al. [40] speculated that golden jackals (*Canis aureus* Linnaeus, 1758) may be a potential host of *B. rossi* in Europe.

South Africa and Nigeria (West Africa) are countries where *B. rossi* in dogs is endemic [3,12,30,31,41,42,43,44]. However, infections have also been detected in dogs in other countries in sub-Saharan Africa (Uganda, Angola, Malawi, Zambia, Kenya, and Sudan), while individual cases have been noted in North America (USA), South America (Brazil), Asia (Singapore), and Europe (France, Germany, Switzerland, Romania, and Russia) [6,40,45,46,47,48,49,50,51,52,53].

The greatest prevalence of infection in dogs has been observed in South Africa and Nigeria (Table 1). In one study, spanning 2000–2006 and incorporating seven of the nine South African provinces, 36.9% of randomly selected domestic dogs from veterinary clinics were found to be infected (420 out of 1138) [41]. The highest prevalences were observed in Mpumalanga province and at the Onderstepoort Veterinary Academic Hospital of the University of Pretoria (Northern Gauteng province and North West province), where 37 of 38 dogs (97.4%) and 355 of 527 dogs (67.3%) were infected with *B. rossi*, respectively. The lowest prevalences were observed in Southern Gauteng, Free State, and Western Cape, where the prevalences amounted to 4.4%, 5.4%, and 6.4%, respectively [41]. In another study, on 126 apparently healthy dogs from four welfare organizations and two townships in the greater Cape Town region (South Africa), *B. rossi* DNA was detected in 16 (12.7%) dogs, while in a separate study in Umkhanyakude District Municipality (KwaZulu-Natal province, South Africa), no *B. rossi* DNA was detected in 49 apparently healthy dogs [54,55]. It should also be noted that, in a retrospective study from the Onderstepoort Veterinary Academic Hospital (Faculty of Veterinary Science, University of Pretoria) in South Africa, 1222 out of 12,706 (9.6%) dogs had babesiosis during the period 2004–2010. However, although *B rossi* is the dominant *Babesia* species in dogs in South Africa, the diagnosis of babesiosis in that study was based on microscopic examination, and the study authors emphasized that 3 to 4% of canine babesiosis may be caused by *B. vogeli* [56]. In another study, on free-roaming, presumably asymptomatic dogs from Zenzele, an informal settlement near Johannesburg (South Africa), seroprevalence amounted to 31.2% (34 of 109 dogs) [57].

A similar prevalence level, 30.8% (33 of 107), was observed in presumably asymptomatic black-backed jackals from two sites in South Africa (Mogale’s Gate Biodiversity Centre and S.A. Lombard Nature Reserve) [35]. Infection prevalence was lower in African wild dogs in South Africa, where only 5.3% (16 out of 301) to 9.6% (5 out of 52) of blood samples collected from probably asymptomatic animals reacted with a *B. rossi* probe on reverse line blot hybridization [37,38]. *B. rossi* DNA was also detected in 4.9% (7 out of 143) of probably asymptomatic cheetahs (*Acinonyx jubatus* Schreber, 1775) from four sites in South Africa, including the Ann van Dyk Cheetah Breeding Centre (De Wildt/Brits and De Wildt/Shingwedzi), the Hoedspruit Endangered Species Centre, and the Cheetah Outreach. In the examined cheetahs, the prevalence of infection was associated with tick burden [58]. However, only a few studies have examined the infection rate of *B. rossi* in *H. elliptica* or other tick species. In one study, *B. rossi* DNA was detected in 2 of 9 (22.2%) tick pools that included *H. elliptica* ticks collected from up to 126 dogs [54]. In another study, van Wyk et al. [64] did not detect *B. rossi* DNA in 231 *H. elliptica* or 361 *R. sanguineus* ticks collected from 61 stray dogs in Potchefstroom, South Africa. This result is surprising as *H. elliptica* is the only confirmed vector of *B. rossi* [65], and in a previous study most of the dogs infected with *B. rossi* were concurrently infested with *H. elliptica* [66]. It should be mentioned that Horak [66] considered *B. rossi* a subspecies of *B. canis*, and in South Africa at that time *H. elliptica* was classified as *H. leachi* [67,68].

The prevalence of *B. rossi* infection in Nigeria has also been reported in several studies. In a study published in 2007, Sasaki et al. [43] detected *B. rossi* DNA in 8 out of 400 (2%) dogs with unknown health status from various regions of Nigeria. In 2011, dogs with tick infestation or clinical signs of tick-borne diseases from four Nigerian states (Kaduna, Plateau, Kwara, and Rivers) were examined, with *B. rossi* present in 6.6% (12 out of 181) of dogs; however, *B. rossi* was only detected in dogs from Plateau State (central Nigeria) and Rivers State (south Nigeria), with prevalence rates of 6.7% and 11.8%, respectively [59]. Another study from Plateau State showed a high prevalence of *B. rossi* infection in sick dogs with presumably clinical signs suggesting tick-borne diseases from Jos city, with 38% (38 of 100) of dogs infected [42]. Despite both studies being undertaken at a similar time in overlapping areas, the results of the studies were considerably different. Kamani et al. [59] conducted their study between August and September 2011, and included 125 dogs from various veterinary clinics in Jos city, finding only 10 dogs (8%) infected with the pathogen. Alternatively, Adamu et al. [42] conducted their study between January and March 2010, and included 100 dogs treated in a veterinary clinic (Evangelical Church Winning All Animal Hospital) in Jos city, finding 38% prevalence of *B. rossi* infection. The differences in study outcomes may result from the relatively small number of animals examined or seasonal variations, and further research on a larger group of dogs is needed to accurately determine the prevalence of *B. rossi* infection in Jos city. In 2017, Takeet et al. [60] determined the prevalence of *B. rossi* in Abeokuta (capital city of Ogun State in southwest Nigeria), revealing that 39 of 209 dogs with unknown health status (18.7%) presented to various veterinary clinics and the Veterinary Teaching Hospital of the Federal University of Agriculture in Abeokuta were infected with the parasite [60]. However, another study from southwest Nigeria (Ibadan in Oyo State) found only 6 of 150 presumably both symptomatic and asymptomatic domestic guard dogs (4%) treated in a private veterinary clinic and the Veterinary Teaching Hospital in University of Ibadan were infected with *B. rossi* [61].

The prevalence of canine babesiosis has also been determined in Zambia, with two studies conducted in Lusaka, Zambia’s capital city [50,69]. However, the prevalence of the first study was based only on microscopic examination, which did not allow for recognition of large *Babesia* species [69], while the second, a molecular study, included only dogs infected with *Babesia* spp. and clinical signs of the infection [50]. The first study was carried out between March 2008 and August 2009 on 1196 dogs from a private veterinary clinic, and also included samples from 361 dogs that presented for rabies vaccination in two low-income residential areas in October and December 2008 (cross-sectional study), along with laboratory records of 7197 sick dogs from the School of Veterinary Medicine (University of Zambia) from October 1994 to December 2009 (retrospective study). The highest prevalence of canine babesiosis in the retrospective study was observed in December (12.4%; rainy season), and the lowest in October (1.6%, dry season). However, the 2008 cross-sectional study revealed that only 0.5% of dogs were infected in the dry season and 0.62% were infected during the rainy season. All recognized *Babesia* parasites in that study were considered as large species [69]. The molecular study was conducted between March 2009 and December 2013 with DNA isolated from 62 *Babesia*-infected dogs. *B. rossi* DNA was detected in 42 dogs (67.7%), with five dogs infected only with *B. rossi* and 37 dogs infected with both *B. rossi* and *B. gibsoni*. The remaining dogs (20 of 62) were infected only with *B. gibsoni* [50]. The combined findings of these studies show that *B. rossi* is not as prevalent in Zambia as it is in South Africa or Nigeria. However, the results are strange, as the first study conducted in Zambia (1994–2009) revealed only large *Babesia* species, while the second study (2009–2013) showed *B. gibsoni* infection (a small *Babesia* species) in 57 out of 62 dogs in the same city of Zambia. These results suggest an outbreak of *B. gibsoni* infection in Zambia after 2009. Another molecular study from Zambia (South Luangwa National Park and Liuwa Plain National Park) did not detect any *Babesia* infections in domestic and wild dogs with unknown health status. However, that study only included a small number of domestic dogs (16) and African wild dogs (22) [62]. A more recent molecular study, published in 2018, revealed low prevalence of *B. rossi* infection in dogs from four cities of Zambia (Lusaka, Mazabuka, Monze, and Shangombo). Only 5 of 247 apparently healthy dogs (2%) were found to be infected with the pathogen, and infections were only detected in two cities: Mazabuka and Shangombo [63]. Surprisingly, despite the inclusion of 50 dogs from Lusaka, the study did not find any *B. rossi* infection among these dogs [63]. This contrasts with previous studies from Lusaka city, which had identified canine babesiosis, with one highlighting the significant contribution of *B. rossi* in the development of disease in affected animals [50,69].

Studies of the prevalence of *B. rossi* have also been conducted in other sub-Saharan African countries. Proboste et al. [45] detected *B. rossi* in 3 out of 38 (7.9%) dogs with unknown health status from rural areas in Uganda. Additionally, *B. rossi* DNA was also detected in one out of nine pools of *H. leachi* ticks collected from dogs in the Mgahinga Gorilla National Park in southwestern Uganda [45]. In a study from Malawi, *B. rossi* DNA was detected in 49 out of 209 (23.4%) apparently healthy dogs from three major cities (Lilongwe, Blantyre, and Mzuzu). However, the majority of *B. rossi* infections (44 cases) were detected in Lilongwe, the country’s capital and largest city [46]. In Kenya, the prevalence of *B. rossi* was determined in 143 sick and apparently healthy dogs from three counties (Nairobi, Mombasa, and Nakuru). Infection was detected in 11 dogs (7.7%), with most cases (8 infected dogs) diagnosed in Nairobi County [47]. A study by Sili et al. [48] covering four communes (Mbave, Sambo, Samboto, and Sede) in the Tchicala-Tcholoanga Municipality of Huambo province in Angola detected *B. rossi* DNA in 20 out of 85 dogs (23.5%), and surprisingly even in one sheep. Lastly, a study carried out in a village (Barbar el Fugara) of eastern Sudan revealed *B. rossi* DNA in 5 out of 78 (6.4%) randomly selected free-roaming dogs with unknown health status between May 1997 and January 2000 [49].

As mentioned above, isolated cases of *B. rossi* infection have been detected in Europe and North America. The first case of *B. rossi* infection in a dog outside of Africa was reported by Fritz [6] in Isère, southeastern France. The study, conducted between March 2006 and March 2008 revealed the dog was also co-infected with *B. canis*. The same study also surprisingly detected *B. rossi* DNA in a horse from Drôme, southeastern France [6]. In 2011, a case of *B. rossi* infection was described in North America, specifically, in Texas, USA. A 5-month-old male Boerboel dog imported from Heilbron, a city in the Free State province in South Africa, exhibited clinical signs of infection such as lethargy, anorexia, coughing, and labored breathing 5 days after arrival. Blood smear examination revealed large *Babesia* parasites, with PCR confirming *B. rossi* DNA in the dog’s blood [51]. In 2020, Birkenheuer et al. [52] published the results of a five-year study (2013–2017) on *Babesia* infection which included over 100,000 dogs from 52 countries across the Americas, Europe, Asia, and Oceania. Single infections with *B. rossi* were detected in Brazil, the United States (co-infection with *B. vogeli*), Singapore, France, Germany, and Switzerland [52]. In a study from Romania in 2019, *B. rossi* DNA was detected in 2 out of 90 dogs (2.2%) that were exhibiting clinical signs of babesiosis such as fever, anemia, thrombocytopenia, icterus, and hemoglobinuria. The remaining dogs (88) were infected with *B. canis* or *B. vogeli* [40]. At a conference in Russia, Egorov et al. [53] reported mixed infections in dogs caused by *B. rossi*, *B. vogeli*, and *B. canis* in the area of the upper Volga River.

Summarizing the studies on the prevalence of *B. rossi* infection, the pathogen is endemic to Africa, and cases detected on other continents result from importation of an infected dog or tick vector. It seems probable that infection is endemic not only in South Africa and Nigeria, but throughout all of sub-Saharan Africa. Gaps in knowledge regarding the occurrence and prevalence of *B. rossi* in countries from this region beyond those mentioned likely result from a lack of studies rather than a lack of the pathogen’s presence in the region.

To provide a more comprehensive picture regarding the transmission and distribution of *B. rossi*, further studies are needed to determine other possible vectors for the pathogen and to confirm whether the *H. elliptica* tick is the only *B. rossi* vector in sub-Saharan Africa.

## 3. Prevalence of *Babesia canis*

*B. canis* is transmitted by the tick *Dermacentor reticulatus* Fabricius, 1794, which occurs in Europe and Asia, and babesiosis caused by this piroplasm species is endemic in many European countries [1]. However, the infection is rare in northern and northeastern Europe, with cases mainly imported [70]. This results from the geographical distribution of the *D. reticulatus* tick, which according to Karbowiak [71] is limited to the Eurasian temperate climate zone between latitudes 50° N and 57° N, and from France and England in the west to the basin of the Yenisey River in Siberia in the east. However, Kjær et al. [72] subsequently reported the presence of *D. reticulatus* in Denmark, southern Norway, and southeastern Sweden, and reports of the tick’s distribution vary. Karbowiak [71] emphasized that *D. reticulatus* ticks also occur north of 39° N latitude, and according to Földvári et al. [73] Portugal and Spain, rather than England and France, represent the western border of the tick’s geographical distribution. While Rubel et al. [74], in their literature study on *Dermacentor* spp. in Europe, did not refer to *D. reticulatus* in Italy, the southernmost foci of *D. reticulatus* in this country is reported to be located in Lombardy (northern Italy, 45° N) in the Groane Regional Park and the Ticino Valley Lombard Park [75]. As reported by Földvári et al. [73], according to the European Centre for Disease Prevention and Control (ECDC) and the Vector-Net project, this tick species even occurs in southern Portugal (38° N); however, the authors expressed concern regarding false data that can be found in those databases [73]. According to Rubel et al. [76], the current geographical distribution of *D. reticulatus* ranges between –9° and 88° E longitude, and between 39° N and 60° N latitude, and includes the south of Russia, and northern and eastern parts of Kazakhstan. However, Livanova et al. [77] detected *D. reticulatus* in dogs from Krasnoyarsk, Russia (92°51′9″ E), and acarological studies have highlighted a permanent increase in this parasite’s geographical range [78,79,80,81,82,83]. According to Mierzejewska et al. [84], this increase in geographical range is associated with the loss of forest areas. It should be mentioned that *D. reticulatus* has a patchy and mosaic distribution with separated foci. The parasite does not occur in the dry Mediterranean climate zone, the cold regions of northern Europe (the northern parts of the British Islands, Scandinavia, and the northern Baltic region), or in the higher mountain regions [73]. However, Hornok and Farkas [85] reported questing *D. reticulatus* ticks in the Mátra Mountains of Hungary at 900–1000 m above sea level.

The role of another tick species, *Ixodes ricinus* Linnaeus, 1758, in the transmission of *B. canis* in dogs is unclear. Although the pathogen has been detected in *I. ricinus* ticks [86,87], the tick is not considered a vector of *B. canis* infection [1,88]. In a study from Latvia, the prevalence of *B. canis* was higher in *I. ricinus* ticks (0.91%, 35 infected ticks out of 3840) than in *D. reticulatus* ticks (0.34%, 2 infected ticks out of 595) [89]. Additionally, Liberska et al. [90] detected *B. canis* DNA in questing larvae, nymphs, and adult male and female *I. ricinus* ticks, showing that *B. canis* can be transmitted transovarially and maintained transstadially in this tick species. Therefore, it cannot be excluded that *I. ricinus* plays a role in the transmission of *B. canis* to dogs; however, the role of *I. ricinus* as a vector of *B. canis* has yet to be proven.

*B. canis* DNA has also been detected in other tick species such as *R. sanguineus*, *Ixodes hexagonus* Leach, 1815, and *Dermacentor marginatus* Sulzer, 1776. Although these were mostly single cases, with the ticks collected from dogs [91,92], Cassini et al. [93] showed transovarial transmission of *B. canis* in *R. sanguineus* ticks, indicating transmission of *B. canis* from the tick to its offspring.

The infection rate of *B. canis* in *D. reticulatus* ticks collected from vegetation or animals has been reported to range from 0% to over 82% [94,95]. Most studies on the prevalence of *B. canis* in populations of *D. reticulatus* have been conducted in Poland, Slovakia, and Germany. The findings of these and other studies on infection rates in *D. reticulatus* ticks are presented in Table 2 and Table 3. The highest prevalence of infection in *D. reticulatus* ticks was observed in Switzerland in 2011. However, due to the small number of collected ticks, which cannot be representative, it seems probable that the percentage of infected ticks in Switzerland is much lower than the reported 82% [95]. In other countries, the infection rate in *D. reticulatus* ticks was lower than reported in Switzerland, but a larger number of ticks were examined. Results from various studies, which are presented in Table 2 and Table 3, show that in general there is a higher prevalence of *B. canis* infection in *D. reticulatus* populations from central and eastern Europe in comparison to the western part of the continent. This is clearly evident in Poland and Slovakia, where ticks from the western parts of these countries were not infected or had very low infection rates in comparison to eastern areas, where a higher prevalence of ticks were infected with *B. canis*. However, discrepancies between infection rates have been observed between studies. For example, one study reported 21.3% of ticks were infected [96], while other studies undertaken at the same time and in the same region, and using the same detection method, reported infection rates of 0.7% or 2.5% [97,98]. The difference in infection rates between western and eastern tick populations may result from the presence of a non-endemic spatial gap for *D. reticulatus* (known as the central European gap) caused by the last glacial maximum, separating the two tick populations [73,99]. According to Földvári et al. [73], the gap will probably disappear, and Kloch et al. [100] proposed that livestock and humans travelling with their pets, rather than wildlife, likely play the main role in the dispersal of *D. reticulatus*, which is likely to lead to the end of the European gap.

The highest prevalence rates of *B. canis* infection in dogs have been observed in Poland (central and eastern parts), Ukraine, Serbia, Romania, and France (Table 4). High prevalence has also been reported in Russia, where the lowest rate (12%) was in the Rostov Region, and the highest rates (between 50% and 75%) were in the city of Ryazan and the Ryazan Region [142]. In other regions (oblasts) such as Kirov, Moscow, Tyumen, Barnaul, and Pyatigorsk Oblast, the prevalence of canine babesiosis was between 30% to 36%, with most of the infections caused by *B. canis* [142]. The data from Russia indicate that babesiosis is one of the most common diseases among Russian dogs, and in some regions of the country half or even a majority of dogs presenting to veterinary clinics are infected with *B. canis*. However, as the article by Domatskiy [142] and the articles cited therein were published in Russian, the authors of this article could not verify the data due to the lack of a version in English.

High prevalence was observed in the studies from Latvia, Lithuania, Bosnia and Herzegovina, and Northern Italy, but only dogs with clinical signs suggesting babesiosis were included in the studies [152,153,154,174]. Some works (e.g., from Croatia or Southern Italy) showed high seroprevalence, yet this indicates contact with the pathogen in the past, not current infection [158,171].

Birkenheuer et al. [52] reported the occurrence of the pathogen in various countries, though not always the prevalence. That study utilized an electronic database from a global commercial laboratory, and the selection of examined blood samples was not random; therefore, the database may have included more cases in which babesiosis was suspected, and the results only indicated occurrence and not the true prevalence of infection. Additionally, for isolated cases in non-endemic regions, the importation of the parasite cannot be excluded.

A large study by Dwużnik-Szarek et al. [82] showed a 2% prevalence of infection by large *Babesia* sp. in dogs from Poland (1558 cases out of 79,204 tested, both sick and healthy animals), whereas the infection rate in dogs from Poland reported by Birkenheuer et al. [52] was 8.5%. This 8.5% is likely an inflated rate and should not be treated as the actual prevalence rate, due to the likely testing mostly or only of dogs with suspected infections. Similarly, Adaszek et al. [146] reported a prevalence of 19.7%, but the study only included dogs with clinical signs of babesiosis and/or tick infestation. On the other hand, Dwużnik-Szarek et al. [82] recognized cases of canine babesiosis based on light microscopy. This allows for the diagnosis of large *Babesia* infection, but not identification of the species of the parasite. However, since *B. canis* is the only endemic species of canine large *Babesia* in Poland [70,101,146], it is highly probable that all or almost all of the 1558 canine babesiosis cases recorded in the study were caused by *B canis*. Thus, the results of Dwużnik-Szarek et al. [82] likely reflect the real prevalence of *B canis* infection in that country.

Single cases of *B. canis* infection in dogs have been detected in countries and regions where the disease is non-endemic, including Japan, the United States, Iran, Turkey (Southeastern Turkey), Estonia, and Nigeria [52,178,179,180,181]. However, a study from North America identified 18 cases of *B. canis* infection among 9367 blood and tissue samples from presumably symptomatic dogs, despite this part of the world being non-endemic for *D. reticulatus* ticks [5]. Similarly, *B. canis* infections in dogs have been recognized in other places where *D. reticulatus* ticks do not occur, including Finland, southern Italy (Campania region), northwestern Iran, the Punjab province in Pakistan, and the Henan province in China [52,173,176,177,182]. Moreover, high seroprevalence has been observed in kenneled dogs with unknown health status in southern Italy (Strait of Messina), which is also a non-endemic region for the parasite’s vector [171]. Pennisi et al. [171] suggested the possibility of other routes of infection such as vertical, direct, or mechanical transmission playing a role in the spread of the parasite. The authors of that article speculated that cross-reaction with *B. vogeli*, the increasing range of *D. reticulatus*, its importation to non-endemic regions, and potentially other tick species as *B. canis* vectors, should also be considered. For instance, Cassini et al. [93] detected the DNA of *B. canis* in 20 out of 376 *R. sanguineus* ticks collected from dogs in Italy, and the DNA of *B. vogeli* in 2 out of 58 *I. ricinus* ticks collected in the same study. Moreover, *B. canis* has been detected in other intermediate mammalian hosts, and not only other canids such as gray wolves (*Canis lupus* Linnaeus, 1758), red foxes (*Vulpes vulpes* Linnaeus, 1758), or golden jackals, with *B. canis* DNA also detected in the blood of horses, cats, and sheep, the feces of bats, and the blood and tissues of rodents (after experimental oral inoculation), and anti-*B. canis* antibodies have been detected in horses [156,183,184,185,186,187,188]. Thus, it cannot be excluded that other mammals and other tick species may play a role in the transmission of this pathogen in regions where *D. reticulatus* is non-endemic.

## 4. Prevalence of *Babesia vogeli*

This parasite is transmitted by *R. sanguineus*, also known as the brown dog tick. However, the taxonomy of this tick is complicated, and includes at least 17 different species. Together, they are referred to as the *R. sanguineus* group, or *R. sanguineus* sensu lato [189]. This group of ectoparasites has a worldwide distribution, but various species of *R. sanguineus* s.l. occur in different areas of the world. For instance, the tick species *Rhipicephalus turanicus* sensu stricto Pomerantzev, 1940, and *Rhipicephalus rossicus* Yakimov and Kohl-Yakimova, 1911, which belong to the *R. sanguineus* group, occur in southern Europe and Asia, whereas *Rhipicephalus guilhoni* Morel and Vassiliades, 1963, and *Rhipicephalus afranicus* Bakkes, Chitimia-Dobler, Matloa, Oosthuysen, Mumcuoglu, Mans, and Matthee, 2020 (previously classified as *R. turanicus*), which also belong to the *R. sanguineus* group, are Afro-tropical species [190,191,192]. Although *R. sanguineus* s.l. has a wide distribution around the world, the pathogen has not been detected in most European countries with a temperate climate. According to the ECDC [193], *R. sanguineus* s.l. occurs only in southern parts of Europe. However, this tick has also been found in the United Kingdom, Iceland, Poland, and in northern Germany (Berlin) [194,195,196,197]. As mentioned above, the tick species *R. rossicus* and *R. turanicus* are part of *R. sanguineus* group found in southern Europe and Asia. The former occurs in the Eurasian steppe (southern ecoregion of Eurasia) between Ukraine and Kazakhstan, including Tajikistan, Uzbekistan, Turkmenistan, Georgia, Azerbaijan, and Russia, while the latter occurs in southern parts of Europe and Asia, from eastern Spain to western China [191,192]. However, according to Nava et al. [198], *R. turanicus* ticks detected in Western Europe, southern Switzerland, and Africa do not belong to *R. turanicus* sensu stricto.

In North America, *R. sanguineus* s.l. occurs mainly in the southern part of the continent. However, both a temperate lineage (*R. sanguineus* sensu stricto) and a tropical lineage (tropical *R. sanguineus* s.l.) have been detected in the northern United States (Wisconsin, Minnesota, Idaho, and Washington State) [199,200,201]. In both northern Europe and the northern United States, *R. sanguineus* ticks have been imported from the southern regions of these continents [194,195,196,201].

In contrast to South and North America, where both tropical and temperate lineages of *R. sanguineus* are present [198], in Australia only the tropical lineage of *R. sanguineus* s.l. has been detected, despite the country, like the Americas, possessing both tropical and temperate climatic regions [202]. According to Chandra et al. [202], the tropical lineage of *R. sanguineus* occurs exclusively in the western and northern parts of Australia, including the Northern Territory, Queensland, the northern parts of New South Wales, and the western parts of Western Australia. It is thought that the parasite was imported to Australia along with dogs at the end of eighteenth century, and the ticks were likely acquired from Tenerife, South Africa, or Brazil, then further spread in Australia among dogs belonging to nomadic Aboriginal communities [202]. Chandra et al. [202] have shown that the temperate lineage of *R. sanguineus* does not occur in Australia, even in regions with a temperate climate. In 1965, Roberts [203] published a taxonomic study on ticks of the genus *Rhipicephalus* and *Boophilus* (the genus currently considered as a subgenus of *Rhipicephalus*) in Australia, proposing a geographical distribution for these ticks and identifying *R. sanguineus* and *Boophilus microplus* Lahille, 1905, as the only two genera present in the country. As the tropical lineage of *R. sanguineus* is the only tick from the *R. sanguineus* group detected in Australia, Chandra et al. [202] proposed the name *R. sanguineus* sensu Roberts (1965) for the Australian brown dog tick. However, in 2021, Šlapeta et al. [204], using material from Australia, identified the tropical lineage of *R. sanguineus* as *Rhipicephalus linnaei* Audouin, 1826. This tick species was initially described in 1826 in Egypt by French entomologist Jean Victor Audouin, who classified the tick species as belonging to the genus *Ixodes* and named it “*Ixodes Linnæi*” (Ixode de Linné). In 2022, *R. linnaei* was officially removed from the *R. sanguineus* s.l. group and considered a separate species [205]. In a study performed from 2012 to 2015, *B. vogeli* was detected in *R. sanguineus* s.l. ticks in Australia, and *B. vogeli* infection is endemic to the country [206,207,208,209].

It is now evident that *B. vogeli* is transmitted not only by *R. sanguineus* s.l. but also by *R. linnaei* ticks, not only in Australia but also in other regions where both *B. vogeli* and ticks previously described as the tropical lineage of *R. sangiuneus* are endemic. Moreover, it cannot be excluded that other species of the *Rhipicephalus* genus can transmit *B. vogeli*; for example, the tick from the “southeastern Europe” lineage of *R. sanguineus* s.l. found in Israel and Egypt has been identified as *Rhipicephalus rutilus* Koch, 1844, and was previously described by Koch in 1844 [210].

It should be mentioned that *B. vogeli* DNA has also been detected in ticks of the genera *Dermacentor*, Haemaphysalis, and *Ixodes*. As mentioned earlier, DNA of *B. vogeli* has been detected in two *I. ricinus* ticks collected from dogs in Italy [93]. The pathogen has also been detected in *D. reticulatus* adult ticks collected from vegetation in Germany and The Netherlands [211], and Zheng et al. [212] detected the parasite in a tick Haemaphysalis flava Neumann, 1897, collected from a mammal of the Erinaceidae family in Jiangxi province in eastern China. In cases of *B. vogeli* DNA found in *H. flava* and *I. ricinus* ticks, it is possible that the ticks had ingested blood containing pathogen DNA that had earlier been injected by another tick species. However, questing *D. reticulatus* adult ticks should be infected as nymphs or larvae, and these stages of this tick species are parasites of smaller mammals like rodents, which are not hosts of *B. vogeli* [73]. Thus, this indicates that transovarial transmission of *B. vogeli* is possible in *D. reticulatus* ticks. However, Sprong et al. [211] detected *B. vogeli* in *D. reticulatus* ticks using high-throughput microfluidic real-time PCR (which accelerates the PCR processes), but did not confirm the infection using conventional PCR or the traditional quantitative PCR. Thus, it seems probable that the detection of *B. vogeli* DNA in questing *D. reticulatus* adult ticks using microfluidic tools was a false positive result.

The prevalence of ticks infected with *B. vogeli* varies in different countries. The infection rate in ticks collected from animals or environment is reported to be very low in Australia and southern Eurasia (Table 5). However, in southern France and southern China (Guangxi province) the prevalence of infection in ticks collected from dogs has been reported as approximately 10% and 12%, respectively [138,213]. The highest prevalence of infection in ticks collected from dogs has been observed in northern Algeria, at 13% [214]. Some studies have reported very high infection rates, but the number of collected and examined ticks was small and not representative. For instance, in central China (Chongqing municipality), 25% of examined ticks were infected with *B. vogeli*; however, only sixteen ticks were examined [213]. Only a few studies have been undertaken in Latin American countries, mainly Brazil, where reported infection rates vary from 1.3 to 3% [215,216]. To the best of the authors’ knowledge, there are no studies on the prevalence of *B. vogeli* infection in *R. sanguineus* s.l. in North America, except for one study from Mexico. However, both *R. sanguineus* group ticks and *B. vogeli* infections in dogs have been detected in the United States and some Caribbean countries, and *B. vogeli* infection was also detected in dogs from Canada [52,201,217]. In the Mexican study, only eighteen ticks were examined and three of them were infected with *B. vogeli* [218]. The only study undertaken in the United States was carried out on Guam Island in Oceania, where one out of 75 ticks was infected with *B. vogeli* [219].

The highest prevalences of *B. vogeli* infection in dogs (sick, healthy, and with unknown health status) have been observed in Australia, Cambodia, Thailand, Egypt, and Costa Rica, particularly in free-roaming dogs or dogs in animal shelters (Table 6). In some countries, although the prevalence was very high, the number of examined animals was low. For instance, in Portugal, only fourteen blood samples from dogs infected with *Rickettsia* spp. were examined with five infected with the parasite; similarly, in Iran, just 40 asymptomatic dogs were included in the study with ten of them infected [236,237]. High prevalences, about 10% or higher, have been observed in Brazil (southern, southeastern, and northeastern parts of the country), Colombia (the northern part of the country), France (the southern part of the country), South Africa (Free State province), Nigeria (the central part of the country), Taiwan, China (the eastern part of the country), and southern India [41,138,226,233,238,239,240,241,242,243,244,245].

Birkenheuer et al. [52] examined only suspected *Babesia* cases; however, their reported *B. vogeli* prevalences were still much lower than the studies cited in, for example, Brazil, Colombia, France, and Australia. A possible explanation for these discrepancies may be locally high prevalences in certain regions of these countries, with the material collected by Birkenheuer et al. [52] coming from various regions including those with low prevalence. For example, high *B. vogeli* infection prevalence has been reported in dogs from northern regions of Australia, but very low prevalences occur in eastern parts of the country [207,208,209]. Similarly, there were high prevalences in eastern and southeastern China in the cities of Taixing and Shenzhen, respectively, but there was a low prevalence reported in Gansu province in the north of the country [233,253,254]. Interestingly, while Hong Kong is in the same region as Shenzhen city, a low prevalence of infection was observed [256]. The time of sampling and differences in the number of tested samples are a possible explanation for the different rates observed from Shenzhen and Hong Kong.

The prevalence of *B. vogeli* infection in dogs is often associated with the infection rate in tick vectors. For example, this was seen in southern France, where 10.5% of ticks and 13.6% of symptomatic dogs and asymptomatic dogs from kennels were infected with the pathogen during the same period [138]. However, in some studies, high prevalences have been observed in dogs while low or moderate infection rates were detected in ticks. For example, in Taixing city in eastern China, *B. vogeli* infection was detected in 11.3% of apparently healthy dogs, but only 3.4% of ticks [233]. The influence of other factors, for example, the use of acaricides to prevent tick infestation, may explain these differences.

It is worth mentioning that *B. vogeli* infection has also been detected in other carnivores from the Canidae and Felidae families. Infection with the parasite was recognized in wild canids in the United States and China, in coyotes (*Canis latrans* Say, 1823) and red foxes (*V. vulpes*), respectively [255,277]. Alternatively, in Argentina, Millán et al. [298] examined 48 blood samples from presumably asymptomatic South American gray foxes (*Lycalopex griseus* Gray, 1837) and one sample from the Andean fox (*Lycalopex culpaeus* Molina, 1782), yet did not detect any *Babesia* sp. DNA in these canids. However, as only 49 samples were tested, the possibility of infection in the American gray fox or the Andean fox cannot be excluded. The pathogen has also been detected in stray cats (most of the animals in good health condition) in Thailand [300], with *B. vogeli* DNA detected in 1.4% (21 out of 1490) of stray cats from Bangkok [300]. In a study from Brazil (Rio de Janeiro State), 2.4% (6 out of 250) of domestic cats with various diseases were infected with this pathogen [301]. In Zimbabwe, Kelly et al. [302] detected *B. vogeli* DNA in felids such as lions (*Panthera leo* Linnaeus, 1758), servals (*Leptailurus serval* Schreber, 1776), and Southern African wildcats (*Felis lybica cafra* Desmarest, 1822), and most of these animals had clinical signs of infection. While the most prevalent infection was caused by *Babesia leo* Penzhorn, Kjemtrup, López-Rebollar, and Conrad, 2001, other tick-borne pathogens were also evident in these animals (*Hepatozoon felis* Baneth, Sheiner, Eyal, Hahn, Beaufils, Anug, Talmi-Frank, 2013, *Cytauxzoon manul* Reichard, Van Den Bussche, Meinkoth, Hoover, and Kocan, 2005, *Theileria* spp., and pathogens of the family Ehrlichiaceae), while five of six Southern African wildcats, and one of two servals, were infected only with *B. vogeli* and exhibited clinical signs of the infection [302]. This indicates that these felids not only harbored *B. vogeli* DNA injected by a tick, but also suffered from feline babesiosis caused by this pathogen. The prevalence of infection caused by *B. vogeli* in these felids was as follows: 11.6% in lions (10 out of 86), 100% in Southern African wildcats (6 out of 6), and 100% in servals (2 out of 2) [302]. However, it is clear that the number of examined Southern African wildcats and servals was too low to calculate accurate prevalences. Kelly et al. [302] also examined four cheetahs (*A. jubatus*); however, the only infection detected in these felids was caused by *B. leo* (4 out of 4 cheetahs were infected). The number of examined cheetahs was too low for any speculations about the possibility of *B. vogeli* infection in these cats. Before further discussion of *B. vogeli* infections, it is worth mentioning a study by Krücken et al. [303] examining vector-borne pathogens in brown and spotted hyenas from Namibia and Tanzania. They identified a DNA sequence similar (similarity 96.9%) to the small subunit ribosomal RNA gene of *B. vogeli* in the blood of one presumably asymptomatic spotted hyena (*Crocuta crocuta* Erxleben, 1777) from Tanzania. The authors suggested that this could represent an unknown species from the *Babesia* genus or a subspecies of *B. vogeli* [303]. As such, it is worth considering that *B. vogeli* may infect not only carnivores of the Canidae and Felidae families, but also animals of the Hyaenidae family.

Despite numerous studies on the prevalence of *B. vogeli* in dogs and ticks across different continents and countries, there are still gaps in knowledge from many regions and countries. For instance, although high prevalences of the infection have been reported in South America, there are no studies from Uruguay, Bolivia, Guyana, French Guiana, Suriname, or Panama, where studies have focused mainly on leishmaniosis and trypanosomosis in dogs [304]. Another example of existing global gaps in the knowledge about *B. vogeli* infection is Namibia and Botswana. Despite confirmation of *B. vogeli* infection in a dog from Namibia, the presence of *R. sanguineus* s.l. in the country, and studies on the prevalence in neighboring countries such as South Africa, Angola, and Zambia [41,63,269,270,305], to the authors’ knowledge there are no studies on the molecular prevalence of *B. vogeli* infection in dogs from Namibia. Similarly, in neighboring Botswana, only one study on the prevalence of canine tick-borne infection has been carried out and it did not show any specific *Babesia* infection in dogs [306]. However, it should be noted that the study only included eighty dogs and all were from one city (Maun) in northern Botswana [306]. Nevertheless, as the data from Table 5 and Table 6 show, *B. vogeli* infection is the most prevalent of the large canine *Babesia* species. This is attributed to the *B. vogeli* tick vector having a broader geographical range in comparison to ticks transmitting *B. canis* or *B. rossi*. Moreover, *R. sanguineus* s.l. is a monotropic tick, meaning the larva, nymph, and adult parasitize the same host species, with the domestic dog the parasite’s main host [307]. In contrast to one-host monotropic cattle ticks of the genus *Rhipicephalus* (e.g., *R. microplus*, *Rhipicephalus annulatus* Say, 1821, or *Rhipicephalus decoloratus* Koch, 1844), which feed not only on the same host species but also on the same individual host (the juvenile and adult stages feeds on the same individual) [308], *R. sanguineus* is a three-host tick [307]. This means that each active developmental stage feeds on a different host, and since it is a monotropic tick, every active tick stage feeds on a different dog [307]. Additionally, transovarial transmission of *B. vogeli* in *R. sanguineus* [229] may promote survival of the infection foci, as the infection may be transmitted to dogs by larvae, nymphs, and adult ticks. Moreover, it seems that subclinical infection in adult dogs may also have a positive influence on the prevalence of *B. vogeli*, as infected dogs without clinical signs may not be treated with babesicidal drugs [34]. According to Penzhorn [34], *B. vogeli*, in comparison to *B. canis* and *B. rossi*, may have the longest evolutionary association with domestic dogs, which may explain the mild or subclinical infections that occur in these animals.

## 5. Prevalence of *Babesia coco*

The first case of canine babesiosis caused by the unnamed *Babesia* species referred to as *Babesia* sp. “Coco” was detected in the United States (North Carolina) in May 2002 [8]. The name was derived from the name of the dog, “Coco”, from this first case report [309]. As mentioned in the introduction section, while the name *Babesia coco* is unofficial, some authors use it as an official species name [5,310].

In 2010, Sikorski et al. [10] published a study which identified 7 dogs infected with an unnamed *Babesia* species. Determination of the 18S rRNA gene sequences revealed the same sequence as previously obtained from the dog in North Carolina in 2002. All dogs were residents of the eastern United States, with five from North Carolina, one from New York, and one from New Jersey. Four of the dogs had a history of travelling to other eastern states including Pennsylvania, Wisconsin, Massachusetts, Kentucky, Ohio, and South Carolina [10]. In addition to babesiosis, all the dogs, including Coco in 2002, had a concurrent noninfectious disease. Six of the dogs had been splenectomized, and two had undergone chemotherapy due to lymphoma; it is noteworthy that all eight dogs were immunosuppressed [8,10]. Similarly, in two other *B. coco* infection case reports, the dogs had undergone chemotherapy because of either lymphoma or adenocarcinoma [9,11].

As discussed by Sikorski et al. [10], it is unclear whether dogs are the pathogen’s primary host, with infection causing disease only in immunosuppressed animals (while other dogs experience subclinical infections), or if dogs are being exposed to a pathogen typically associated with another host species, with infection persisting and leading to disease only in immunosuppressed dogs. However, Dear and Birkenheuer [309], based on unpublished data, suggested that about 25% of dogs infected with *B. coco* were not immunocompromised and that the pathogen may be the etiological agent in some cases of a fever of unknown origin.

The pathogen was detected in five ticks *Amblyomma americanum* Linnaeus, 1758 (lone star tick), collected between 2005 and 2012 from a dog, a feral pig, and humans from four southern and eastern states of the United States [311]. Dear and Birkenheuer [309] speculated that the *A. americanum* tick may be a vector for *B. coco*, based on the fact that all cases of *B. coco* infection have been detected within the geographical distribution of *A. americanum*.

A study of ticks and tick-borne pathogens in recreational greenspaces in Gainesville (Florida) conducted by Bhosale et al. [312] in 2021 collected questing ticks of six species belonging to various genera, including *Amblyomma* (*A. americanum*, *Amblyomma maculatum* Koch, 1844), *Dermacentor* (*Dermacentor variabilis* Say, 1821), *Haemaphysalis* (*Haemaphysalis leporispalustris* Packard, 1869), and *Ixodes* (*Ixodes affinis* Neumann, 1899, *Ixodes scapularis* Say, 1821); however, *B. coco* DNA was detected only in *A. americanum*. The infection rate was very low, only 7 out of 1076 lone star ticks were infected with the pathogen [312]. Similarly, Noden et al. [313] found that only 22 out of 4714 questing lone star ticks collected in 2017 and 2018 in Oklahoma City were infected with *B. coco*. The pathogen was not detected in either *D. variabilis* or *A. maculatum* ticks, which were also collected in the study [313]. The findings from Bhosale et al. [312] and Noden et al. [313] support the previous supposition that *A. americanum* ticks are the vector of *B. coco*.

According to the authors’ knowledge, there is only one study in which the prevalence of *B. coco* infection in dogs has been determined. A study by Barash et al. [5], performed between June 2015 and June 2018 by the Canine Vector-Borne Disease Diagnostic Panel of the Vector-Borne Disease Diagnostic Laboratory (North Carolina State University), found only 0.17% (16 out of 9367) of dogs were infected with *B. coco*. Importantly, as the study authors indicated, prevalence was determined from samples submitted to the Vector-Borne Diagnostic Laboratory, and most of the dogs included likely had clinical signs suggesting a vector-borne disease [5]. Thus, the true prevalence of infection remains unknown, but is probably lower than 0.17%.

Based on the results of Sikorski et al. [10] and Barash et al. [5], and the lack of similar findings from elsewhere in North America, it appears that *B. coco* infection primarily occurs in the Eastern Atlantic United States. Infection was also detected in a dog from Texas; however, the dog had a history of travelling to various southeastern and eastern states [9]. As mentioned above, *A. americanum* ticks infected with *B. coco* have been collected in Georgia, Kentucky, Tennessee, and Texas [311]. Thus, it seems probable that the infection caused by *B. coco* can occur in the southeastern and eastern United States, corresponding with the distribution of the lone star tick [314].

Isolated infections with *B. coco* have been detected in other animal species. Shaw et al. [4] detected *B. coco* DNA (99% similarity) in the American black bear (*Ursus americanus* Pallas, 1780) during a study on the prevalence of *Babesia* spp. in 201 presumably asymptomatic black bears in northwestern New Jersey. Although high prevalence of *Babesia* infection was observed among examined animals, most were infected with small piroplasms, mainly *Babesia* sp. AJB-2006 and *Babesia microti* França, 1912, and *B. coco* infection was recognized in only one bear [4]. In a study on the prevalence of protozoan parasites in small and medium mammals from East Texas, *Babesia* infection was detected in five out of fifteen presumably asymptomatic raccoons (*Procyon lotor* Linnaeus, 1758), with one racoon co-infected with *B. microti* and *B. coco* [315].

In a case report of co-infection caused by *Babesia* sp. and *Cytauxzoon felis* Kier, 1979, in a bobcat (*Lynx rufus* Schreber 1777) with unknown health status from the state of Georgia in the United States, Shock et al. [316] detected a piroplasm closely related to *B. coco* (92% similarity). The authors reported only one difference in the nucleotide sequences of the internal transcribed spacer 1 region between *B. coco* and the *Babesia* sp. detected in the bobcat, specifically a 45 base pair (bp) insertion at nucleotide site 434 in the obtained 601 bp PCR product, with the rest of the sequence identical to the GenBank sequence (EU109720) from nucleotides 1 to 557 [316]. However, it should be noted that the EU109720 DNA sequence is longer, at 1002 base pairs. Shock et al. [316] speculated that the *Babesia* sp. from the bobcat may be a variant of *B. coco*. Moreover, in May 2012, two of the report’s four authors (Shock, B., and Yasbley, M.) submitted this 601 bp sequence to Genbank under accession number JX021526; however, in the article of Shock et al. [316], the presented phylogenetic tree mislabeled the *Babesia* sp. from the bobcat as accession number AY618928. Accession number AY618928 actually refers to the first case of *B. coco* infection recognized in May 2002 [8]. Therefore, it is not clear to the authors if the *Babesia* sp. detected in the bobcat from Georgia can be considered as *B. coco* without further study.

To summarize the studies on *B. coco* infection, the pathogen occurs in the eastern and southeastern United States. The lone star tick (*A. americanum*) is the only known vector for this pathogen. The prevalence of infection in dogs remains unknown but appears to be relatively low, and it may reflect the low infection rate in *A. americanum* ticks. It is still unclear if the infection occurs only in immunosuppressed dogs or if non-immunosuppressed dogs are also infected but clinical signs of infection are only observed in immunosuppressed dogs. The name *B. coco* is still unofficial, and the pathogen remains unnamed. This results from the fact that the authors who originally detected this infection in a dog from North Carolina in 2002 and submitted the DNA sequence to GenBank [8] considered it inappropriate to name this pathogen, considering that the DNA sequences of over 100 named *Babesia* spp. remained undetermined [11]. It cannot be excluded that a previously named *Babesia* species that parasitizes non-canine animal species may share the same DNA sequence as *B. coco*, and consequently that the original name of the pathogen should be used as an official name instead of *B. coco*. Nonetheless, the authors of this review article considered that using *B. coco* as an official name with proper explanation would be clearer for readers.

## 6. Risk Factors for Large *Babesia* Infections

In contrast to small *Babesia* spp. infections in dogs (*B. gibsoni*, *B. conradae*, or *B. vulpes*), which can be transmitted by both the tick vector and presumably by dog bites during fights with other dogs or canids (some studies indicate this especially occurs with male dogs) [5,276,317,318,319,320], infections caused by large *Babesia* spp. do not appear to be transmitted via dog bites. Instead, large *Babesia* spp. infections are transmitted by ticks, and the geographical distribution of tick vectors limits geographical occurrence [321]. Fighting between dogs is not considered a risk factor for large *Babesia* spp. infection. Infections caused by both small and large *Babesia* spp. can also be transmitted vertically (from mother to offspring) or through blood transfusion [1,88].

Based on the higher frequency of *B. rossi* infections in mixed-breed dogs, Maltese poodles, Staffordshire bull terriers, Rottweilers, and Bull Terriers in South Africa [12], Mellanby et al. [56] hypothesized that Toy breeds have a lower risk of babesiosis than working dogs in South Africa. Subsequently, Toy breeds (Chihuahua, Maltese, Pekingese, Pomeranian, Pug, Yorkshire Terrier), some Terriers (Bull Terrier, Jack Russel Terrier, Staffordshire Bull Terrier), and some other breeds (e.g., smooth-haired Dachshund, miniature Doberman, and Bulldog) were found to have a lower risk of the disease in comparison to Labrador Retrievers, which acted as the reference breed. Among working dogs, Siberian huskies were found to have the highest risk of infection (odds ratio (OR) amounted to 1.72) [56]. The authors also observed a higher risk of the disease in males (both intact and neutered) and neutered females, in comparison to intact females [56].

In a study on *B. canis* infection in Italy, Cassini et al. [93] did not observe an increased risk of infection with the pathogen in male dogs in comparison to females. However, in that study both spayed and intact females were included in the female reference group. The study did report an increased risk of babesiosis in kenneled dogs in comparison to companion animals (OR = 4.342), and in dogs aged between 25 and 48 months in comparison to dogs aged 0–24 months old (OR = 1.722) [93]. A study undertaken in Poland showed a higher risk of infection with *B. canis* in rural dogs compared to urban dogs (OR = 1.7), and in purebred dogs (mainly German Shepherd Dogs, Irish Setters, and American Staffordshire Terriers) compared to mixed-breed dogs (OR = 2.24) [146]. The highest risks of infection were associated with location in eastern Poland (OR = 8.91), and previous infection with *B. canis* (OR = 17.9). The use of acaricides was found to be a protective factor against infection (OR = 0.32) [146]. Considering eastern Poland is a region endemic for canine babesiosis [82], it is evident that the risk of infection is higher in that part of Poland. Moreover, the high risk of infection in dogs that have previously been infected with the pathogen may be associated with other factors such as an endemic region, rural areas, and the lack of treatment with acaricides. The authors of the study also mentioned a higher risk of infection in male dogs and in dogs younger than 12 months [146]. However, the authors did not report *p*-values, only 95% confidence intervals, and for these two variables (sex and age), the lowest values of the confidence intervals were less than 1, and the highest values were above 1 [146]; therefore, these two variables should not be considered as risk factors of *B. canis* infection in Poland.

An epidemiological study conducted in Croatia examining the seroprevalence of *B. canis* infection showed an increased risk for seropositivity to *B. canis* among dogs older than 3 years in comparison to dogs younger than 12 months (OR > 10). Additionally, hunting dogs (OR = 4.57) and outdoor/shelter dogs (OR = 2.56) were more likely to be seropositive in comparison to companion indoor dogs [158]. Similarly, a study in Romania also identified a higher risk of seroreactivity in hunting dogs, with an odds ratio identical to that found in Croatia (OR = 4.57) [322].

A study from southern Italy utilized PCR to identify both *B. canis* and *B. vogeli* infections among dogs; however, risk factors for infection were based on *B. canis*/*B. vogeli* seroprevalence [173]. An increased risk for seropositivity was identified in male dogs (OR = 1.85) and in dogs with long coats (OR = 1.61), in comparison to female and short-haired dogs, respectively. The authors also reported that the risk for seroreactivity increased with age (OR = 1.01) and was higher in the Salerno province (OR = 1.71) [173]. However, the study did not provide *p*-values, only 95% confidence intervals, for the presented odds ratios, and did not indicate the reference variables. Thus, the reference variables must be inferred (e.g., female dogs or short-haired dogs) and the statistical significance can be found only in a table comparing seroprevalence between various groups (e.g., males and females). The article did indicate statistical significance (*p* < 0.05) for variables such as dog age, coat length, gender, and province [173]. Thus, while the mentioned risk factors for seroreactivity were statistically significant, interpretation is hindered due to how the results were presented.

In a study conducted in Nigeria, the wet season, which is associated with increased tick activity, was linked to an increased risk of infection with *B. vogeli* in comparison to the dry season (OR = 2.08) [244]. Similar observations have been made in Europe, where most *B. canis* infections have been detected in dogs during spring and autumn [143,323]. The study in Nigeria also revealed that the risk of infection was lower in dogs younger or older than dogs in the 12-to-36-month age group [244]. Although the authors of that study indicated other risk factors (e.g., male sex or various exotic dog breeds) [244], those parameters were not statistically insignificant (*p*-values higher than 0.05), and therefore cannot be considered as risk factors for *B. vogeli* infection in Nigeria. A study from Egypt on the prevalence of *B. vogeli* infection in 275 dogs showed that the higher risk of infection in male dogs was not statistically significant [267]. The only significant risk factors for infection in dogs from Egypt were the soil type of the floor in dog shelters (OR = 6.1) in comparison to paved floors, and tick infestation (OR = 3.8) in comparison to a lack of tick infestation [267]. However, a study from Brazil did not find an association between the type of the floor where dogs were kept and the frequency of infection with the parasite [241]. Another study from northern Brazil found increased risks for seropositivity for *B. vogeli* included medium-sized dog breeds (OR = 2.98), contact with Caatinga (a region with small, thorny trees; ecoregion in Brazil) or forest (OR = 2.22), and access to streets (OR = 1.56) [216]. In Brazil, animal age is also a risk factor for seroreactivity to *B. vogeli.* Dogs between 2 and 5 years old have a 2.66 times (OR = 2.66) increased risk of *B. vogeli* seropositivity, and dogs older than 5 years have a 4.3 times increased risk when compared to dogs younger than 2 years old (OR = 4.3) [293]. This is to be expected, as older dogs have had a longer time to be exposed to *B. vogeli* than younger animals. The presence of ticks increased the risk of seropositivity (OR = 3.12), and interestingly this risk was higher in dogs infested with *Amblyomma cajennense* Fabricius, 1787 (OR = 3.06), in comparison to dogs without a tick infestation. In dogs infested with *R. sanguineus* s.l., the result was statistically insignificant [293]. These results for tick species infestation may be incidental, as seroprevalence but not molecular prevalence was studied, and such a study reflects previous contact with the pathogen, not current exposure. However, other research from Brazil examining the molecular prevalence of *B. vogeli* infection showed that dogs infested with ticks, dogs younger than 5 years old, and dogs without access to a shelter were all at higher risk of infection (OR = 2.22, 2.12, and 2.08, respectively) [239]. Surprising results from a study in western Brazil showed that none of the variables of breed, age, sex, tick infestation, indoor or outdoor living, or the use of the acaricides were associated with increased or decreased risk of *B. vogeli* infection [324]. Similar results were observed in a molecular study from eastern Brazil (Pernambuco State), where none of the examined variables (sex, age, breed, rural/urban area, and infestation with ticks including *R. sanguineus* s.l.) were statistically significant risk factors for *B. vogeli* infection [291]. These discrepancies between various studies on *B. vogeli* infection risk factors may result from subclinical infections in many dogs. For example, another study from Pernambuco State in Brazil reported a high seroprevalence (57.9%) among 404 examined dogs [216]. Therefore, a study utilizing both molecular and serological approaches on a much larger group of dogs may provide a more conclusive understanding of the risk factors for *B. vogeli* infection, at least in Brazil or South America.

To the authors’ best knowledge, there are no studies that have determined the risk factors for *B. coco* infection. The main risk factor for babesiosis caused by this pathogen appears to be immunosuppression [8,9,10,11]. However, it is not clear if the immune status of the host increases the risk of infection or the risk of disease development.

Various studies have shown that the treatment of dogs with acaricides can reduce the risk of *Babesia* transmission from infected ticks [325,326,327,328,329]. In a study from Italy, dogs that underwent regular treatment with acaricides had a decreased risk of infestation with ticks infected with *Babesia* spp. or *Theileria* spp. in comparison to dogs without such treatment (OR = 0.24); additionally, dogs from urban areas had a lower risk in comparison to dogs from rural and forest habitats (OR = 0.31) [224]. Thus, the lack of regular acaricide use and living in rural or forest regions can be considered risk factors for infestation with ticks infected with piroplasms such as *Babesia* spp. or *Theileria* spp. The study also showed that kennels were not associated with increased risk of tick infestation in comparison to indoor housing [224].

It is also worth mentioning the risk of *Babesia* spp. infection resulting from blood transfusion. Such infections in dogs, caused by both large and small piroplasms, have been observed [330,331]. However, the use of proper clinical and laboratory procedures, as described in detail by Wardrop et al. [332] and Nury et al. [333], can minimize any risk of infection transmission by blood transfusion. In a study undertaken by Nury et al. [333] between 2010–2016, no *Babesia* spp. DNA was detected in 6140 blood units from the Canadian Animal Blood Bank. However, DNA from *Anaplasma phagocytophilum*, *Bartonella* spp., *Brucella canis*, *Mycoplasma haemocanis*, and “*Candidatus* Mycoplasma haematoparvum,” or a combination of these pathogens, was detected by PCR in 1.1% of blood units [333]. That study indicates there is a low risk of infection with various blood-borne pathogens, while emphasizing that following proper clinical and laboratory procedures can further reduce the risk of infection by blood transfusion. However, the study did not answer whether pooling blood for PCR testing can influence *Babesia* DNA detection [333]. This is especially relevant in areas endemic for canine babesiosis.

## 7. Conclusions

Among the four large *Babesia* species, *B. vogeli* is the most prevalent globally. This stems from its wide spectrum of monotropic vector species, the ability to cause predominantly mild or subclinical infections, and its long evolutionary association with dogs. The prevalence of two other large *Babesia* spp. (*B. canis* and *B. rossi*) is limited by their association with specific polytropic vector species such as *D. reticulatus* or *H. elliptica*, with preferences for various hosts at different tick stages (the juvenile stages of both tick species prefer small rodents), and the tendency to cause more severe forms of babesiosis in comparison to *B. vogeli* infection. *B. rossi* is endemic to Africa, with the highest prevalences observed in South Africa and Nigeria, but a likely distribution throughout sub-Saharan Africa, while *B. canis* is endemic in temperate European countries and also found in parts of Asia. The prevalence of *B. coco* infection in dogs is very low and is limited by the low infection rate in its presumptive tick vector (*A. americanum*), the limited geographical range of the vector, and the need for immunosuppression in dogs for infection/disease to occur. However, knowledge about *B. coco* infection and its natural host is also limited, and further study on *B. coco* hosts may provide insight into the reasons for the low prevalence of infection in dogs.

Different risk factors for infection have been reported for the different large *Babesia* species in various countries, although the relationship between risk factors and infection does not appear to be as strongly linked as observed with *B. gibsoni*, where infection is strongly associated with breeds of fighting dogs (American Pit Bull Terriers or Tosa dogs), especially male dogs of these breeds in non-endemic regions. However, different dog breeds and dog sizes have been reported as risk factors for infection (e.g., hunting dogs (*B. rossi*) and working dogs (*B. rossi*) have been observed to be at higher infection risk). Additionally, a higher risk of infection with large *Babesia* spp. in male dogs has also been observed in some studies, although most studies have not shown sex to influence prevalence of infection. Transmission via ticks is the almost exclusive mode of infection for large *Babesia* spp., which is reflected in the most important risk factors for infection being linked to exposure to infected ticks: regions endemic for the infection, living in rural areas, kennels or animal shelters, the season of the year (which is associated with increased tick activity), infestation with ticks, and lack of treatment with acaricides. Alternatively, immunosuppression and living or visiting the eastern or southeastern United States are the only known factors associated with *B. coco* infection.

Despite a range of studies into the prevalence and risk factors associated with large *Babesia* species in various regions of the world, a lack of studies in certain locations and the limitations of other studies have left gaps in the knowledge about these protozoan parasites. For example, the interpretation of risk factors from seroprevalence can be difficult as seroreactivity reflects contact with the pathogen in the past, while the variables used for the calculation of odds ratios represent the current situation of the dog. Additionally, this can skew towards older dogs having a higher chance of seroreactivity as these dogs have had more time to come into contact with the parasite. The varying nature of the diseases caused by these parasites can also limit understanding and comparison between species; for example, the estimation of risk factors for *B. vogeli* infection are also difficult due to the mild or subclinical nature of infections, and clinical studies mainly include dogs that show signs of various diseases, whereas dogs with subclinical infection often do not present to veterinary clinics.

Despite the comprehensive findings presented in this review, it is essential to acknowledge that uncertainties and limitations exist regarding canine large *Babesia* species. It is apparent that further work is required to fill the gaps in the existing understanding of large *Babesia* in canines, including their prevalence and risk factors. This will allow us to ascertain the true impact of infections on dog populations and facilitate improvement in their welfare.

## Figures and Tables

**Table 1:** Prevalence of *Babesia rossi* infection in dogs and in other Carnivora.

Country/Region		Prevalence of Infected Dogs (No. of Infections)	Time of Blood Collection	Ref.
South Africa	South Africa (7 out of 9 provinces)	36.9% (420 out of 1138)	2000–2006	[41]
	Cape Town region	12.7% (16 out of 126)	Before 2014 ^1^	[54]
	Eastern South Africa: KwaZulu-Natal province	0% (0 out of 49)	Before 2020 ^1^	[55]
	South Africa (Onderstepoort Veterinary Academic Hospital, University of Pretoria)	9.6% (1222 out of 12,706) ^2^	2004–2010	[56]
	Zenzele (settlement near Johannesburg)	31.2% (34 out of 109 dogs) ^3^	2008–2014	[57]
	North-central part of South Africa (Mogale’s Gate Biodiversity Centre and S.A. Lombard Nature Reserve)	30.8% (33 out of 107) ^4^	Between 2011 and 2017 ^1^	[35]
	Northern South Africa (provinces: North West and Limpopo)	5.3% (16 out of 301) ^5^	Before 2008 ^1^	[37]
	Northern South Africa (Kruger National Park)	9.6% (5 out of 52) ^5^	Before 2021 ^1^	[38]
	Northeast and Southwest	4.9% (7 out of 143) ^6^	2007–2011	[58]
Nigeria	Various regions of Nigeria	2% (8 out of 400)	2004–2005	[43]
	Central, western, and southern regions of Nigeria (States of Kaduna, Plateau, Kwara, and Rivers)	6.6% (12 out of 181)	2011	[59]
	Central Nigeria (Plateau State: Jos city)	38% (38 out of 100)	2010	[42]
	Southwestern Nigeria (Ogun State: Abeokuta city)	18.7% (39 out of 209)	2014–2015	[60]
	Southwestern Nigeria (Oyo State: Ibadan)	4% (6 out of 150)	2020	[61]
Zambia	Eastern and western parts of Zambia (South Luangwa National Park and Liuwa Plain National Park)	0% (0 out of 40) ^7^	2009–2011	[62]
	Southwestern Zambia: cities of Mazabuka and Shangombo	2% (5 out of 247)	2016	[63]
Uganda	Southwestern Uganda: rural areas near 3 national parks (Bwindi Impenetrable National Park, Mgahinga Gorilla National Park, Queen Elizabeth National Park)	7.9% (3 out of 38)	2011	[45]
Malawi	Southern, central, and northern part of Malawi (cities of Lilongwe, Blantyre, and Mzuzu)	23.4% (49 out of 209)	2018–2019	[46]
Kenya	Southern and southwestern regions of Kenya: Nairobi, Mombasa, and Nakuru counties	7.7% (11 out of 143)	Before 2021 ^1^	[47]
Angola	Central-western Angola: Huambo province (Tchicala-Tcholoanga Municipality)	23.5% (20 out of 85)	2016	[48]
Sudan	Eastern Sudan: village of Barbar el Fugara	6.4% (5 out of 78)	1997–2000	[49]

^1^ Unknown time of blood collection. ^2^ Result based on microscopic examination of blood smear. ^3^ Seroprevalence. ^4^ Prevalence of infection in black-backed jackals (*Canis mesomelas*). ^5^ Prevalence of infection in African wild dogs (*Lycaon pictus*). ^6^ Prevalence of infection in cheetahs (*Acinonyx jubatus*). ^7^ Prevalence of infection in domestic dogs and African wild dogs.

**Table 2:** Prevalence of *Babesia canis* infection in *Dermacentor reticulatus* ticks collected from vegetation in various countries.

Country/Region		Prevalence of Infected Ticks (No. of Infections)	Time of Tick Collection	Ref.
Poland	Western Poland	0% (0 out of 1233)	2016–2018	[101]
	Western Poland	0% (0 out of 592)	2012–2014	[102]
	Southwestern Poland (Wrocław)	0% (0 out of 337)	2013–2014	[103]
	Eastern Poland (Lublin province)	0.7% (4 out of 582)	2011–2012	[97]
	Eastern Poland	5.4% (108 out of 1993)	2011–2014	[102]
	Eastern Poland	5.9% (74 out of 1264)	2016–2018	[101]
	Northeastern Poland (Białystok, Augustów)	6.7% (18 out of 270)	2017–2019	[104]
	Northeastern Poland (Białystok)	7.3% (27 out of 368)	2018	[105]
	Northeastern Poland (The Protected Landscape Area of the Bug and Nurzec Valley)	7% (21 out of 301)	2016–2017	[106]
Germany	Saxony (Leipzig)	0% (0 out of 804)	2009	[94]
	Bavaria	0% (0 out of 135)	2009	[94]
	Bavaria	0.3% (1 out of 301)	2010–2013	[107]
	Saarland	2.5% (10 out of 397)	2008	[108] ^1^
Austria	Eastern Austria	16.7% (1 out of 6)	2007–2008	[109]
Slovakia	Western Slovakia ^2^	0% (0 out of 2999)	2009–2011	[110]
	Southwestern Slovakia	1% (1 out of 100)	2002	[111]
	Western Slovakia	1.8% (11 out of 600)	2011–2012	[112]
	Southwestern Slovakia	2.2% (1 out of 45)	2014	[113]
	Southwestern Slovakia	2.3% (28 out of 1205)	2009	[114]
	Eastern Slovakia	14.7% (48 out of 327)	2009	[114]
	Southern Slovakia	35.6% (116 out of 326)	2004–2010	[115]
United Kingdom	Wales and Southern England	0.3% (1 out of 294)	2019–2020	[116]
	Wales	3.3% (1 out of 30)	2010–2012	[117]
	Southern England (Devon and Essex)	17.1% (14 out of 82)	2010–2016	[117]
Spain	Northern Spain (Basque Country)	1% (1 out of 97)	2003–2005	[86]
Belgium	Belgium	0% (0 out of 282)	2010	[118]
	Belgium	0% (0 out of 289)	2011–2013	[119]
The Netherlands	The Netherlands	3.1% (14 out of 444)	2011–2013	[119]
Latvia	Latvia	0.3% (2 out of 595)	2017–2019	[89]
Latvia and Lithuania	Latvia and Lithuania	1.3% (31 out of 2436) ^3^	2013–2015	[120]
Ukraine	Various regions	0% (0 out of 141)	2018	[121]
	Chernobyl exclusion zone	2.9% (6 out of 205)	2009–2012	[122] ^1^
Czech Republic	Czech Republic	2.8% (22 out of 783)	2018–2021	[123]
Russia	Southwestern Siberia (Novosibirsk)	4.2% (3 out of 72)	2003	[124]
	Southwestern Siberia (Omsk and Novosibirsk)	3.4% (3 out of 87)	2003–2004	[125]
Serbia	Serbia	20.7% (11 out of 53)	2007, 2009	[126]
	Northern Serbia	21.6% (11 out of 51)	2007, 2009	[127]
Hungary	Budapest	8.2% (34 out of 413)	2014–2015	[128]
Italy	Lombardy	10.9% (53 out of 488)	2015–2016	[129]
Switzerland	Swiss Midlands	82.6% (19 out of 23)	2011	[95]

^1^ Discrepancies between results presented in various parts of the article. ^2^ The study performed in western Slovakia and the southeasternmost part of the Czech Republic. ^3^ Ticks infected with *Babesia* spp., only 18 PCR products were sequenced (*B. canis* in 17 ticks, *B. venatorum* in 1 tick).

**Table 3:** Prevalence of *Babesia canis* infection in *Dermacentor reticulatus* ticks collected from animals (mainly dogs) in various countries.

Country/Region		Prevalence of Infected Ticks (No. of Infections)	Time of Tick Collection	Ref.
Poland	Southwestern Poland (Wrocław)	0% (0 out of 46)	2013–2014	[103]
	Central Poland	11% (42 out of 381)	2003–2005	[130]
Germany	Berlin/Brandenburg Region	0% (0 out of 140)	2010–2011	[131]
Slovakia	Southwestern Slovakia	4.8% (1 out of 21)	2014	[113]
United Kingdom	United Kingdom	10% (1 out of 10) ^1^	2015	[132]
The Netherlands and Belgium	The Netherlands and Belgium	0% (0 out of 133)	2007–2013	[119]
The Netherlands	The Netherlands	0% (0 out of 344)	2005–2006	[133]
Latvia	Latvia	14.8% (4 out of 27)	2016	[134]
Ukraine	Various regions	2.1% (6 out of 281)	2018	[121]
	Kiev	6.1% (2 out of 33)	2010	[135]
Hungary	Hungary	29.9% (43 out of 144)	2004–2007	[136]
	Hungary	24.2% (8 out of 33)	Before 2016 ^2^	[137]
France	France	12% (3 out of 25)	Before 2016 ^2^	[137]
	Southern France	9.7% (3 out of 31)	2010–2012	[138]
Switzerland	Lake Geneva Region	20% (3 out of 15)	2005–2006	[139]
Austria	Eastern Austria	33.3% (2 out of 6)	2007–2008	[109]
Russia	Various cities between Smolensk and Krasnoyarsk	20.3% (82 out of 404)	2016	[77]
Serbia	Serbia	17% (8 out of 47) ^3^	2010–2013	[140]
	Northern Serbia: Vojvodina province	33.3% (6 out of 18)	Before 2016 ^2^	[141]
Spain	Spain	10.8–58.8% of pools of ticks	2014–2015	[91]

^1^ Infected tick was fully engorged, collected from a dog which had recently returned from France. ^2^ Unknown time of collection. ^3^ Ticks collected from golden jackals (*Canis aureus*).

**Table 4:** Prevalence of *Babesia canis* infection in dogs and in other canids.

Country/Region		Prevalence of Infected Dogs (No. of Infections)	Time of Blood Collection	Ref.
**Europe**				
Poland	Central Poland (Warsaw)	11.8% (48 out of 408)	2003–2004	[143]
	Central Poland	28% (23 out of 82)	2006–2008	[144]
	Central Poland	30.4% (72 out of 237)	2015–2021	[145]
	Central and Eastern Poland	5.3% (1532 out of 28,881)	2016–2018	[82]
	Western Poland (including areas endemic and non-endemic for *D. reticulatus*)	0.5% (26 out of 50,323) ^1^	2016–2018	[82]
	Poland (16 voivodeships)	19.7% (158 out of 800) ^2^	2008–2010	[146]
	Poland	8.5% (14 out of 165) ^3^	2013–2017	[52]
	Poland (Western and Eastern Poland)	2.4% (9 out of 381) ^4^	2016–2018	[147]
Czech Republic	Southern Czech Republic: South Moravian Region (Břeclav District)	0% (0 out of 41)	2010	[148]
	Southern Czech Republic: South Moravian Region (Břeclav District)	12.2% (5 out of 41) ^5^	2010	[148]
	Southern Czech Republic: South Moravian Region and South Bohemian Region	1.3% (5 out of 377)	2015	[149]
	Czech Republic	10.8% (7 out of 65) ^3^	2013–2017	[52]
Slovakia	Western Slovakia (Malacky District)	0% (0 out of 100)	2010	[150]
	Southern Slovakia (towns: Komárno and Nové Zámky)	20.5% (24 out of 117)	2010–2011	[150]
	Slovakia	3.8% (14 out of 366) ^6,7^	Before 2014 ^8^	[151]
Ukraine	Western Ukraine	29% (45 out of 155)	2015–2021	[145]
	Kiev	26.1% (6 out of 23)	2011	[135]
Latvia	Riga, southern and western Latvia	16.4% (43 out of 262) ^2^	2016–2019	[152]
Lithuania	Central Lithuania: Kaunas	76.4% (94 out of 123) ^2^	2013–2014	[153]
Bosnia and Herzegovina	Sarajevo Canton	85% (68 out of 80) ^2^	2014–2016	[154]
	Bosnia and Herzegovina	0.8% (1 out of 119) ^4^	2013–2014	[155]
Croatia	Western Croatia	6.5% (7 out of 108) ^9^	1996–2015	[156]
	Croatia	2.4% (20 out of 848)	2007–2008	[157]
	Croatia	20% (87 out of 435) ^5^	Before 2017 ^7^	[158]
Serbia	Suburban and rural Belgrade municipalities	13.5% (15 out of 111)	2015	[159]
	Serbia	4.2% (9 out of 216) ^10^	2010–2013	[140]
Slovenia	Central Slovenia: Ljubljana	4.6% (11 out of 238)	2000–2002	[160]
	Slovenia	1.3% (1 out of 77) ^3^	2013–2017	[52]
Albania	Central Albania (Tirana city)	13.3% (4 out of 30)	2008	[161]
Bulgaria	Central Bulgaria (Stara Zagora city)	16.2% (27 out of 167) ^5^	Before 2015 ^7^	[162]
Republic of Moldova	Southern Moldova: Cahul city	11.9% (5 out of 42)	2018–2019	[163]
	Central Moldova: Chișinău city	11.5% (9 out of 78)	2018–2019	[163]
Romania	Southern Romania	7% (21 out of 300)	2017	[164]
	Southern Romania: Ilfov County	29.2% (28 out of 96)	2013–2014	[165]
	Romania	37.6% (41 out of 109) ^11^	2009–2010	[166]
	Romania	8.9% (5 out of 56) ^10,12^	2013–2015	[167]
Hungary	Hungary	5.7% (37 out of 651) ^5^	2005	[168]
	Hungary	50% (39 out of 78) ^11^	2009–2010	[166]
	Southwestern Hungary: Somogy County	5.5% (5 out of 90)	2017	[7]
Austria	Austria	9.9% (113 out of 1146) ^3^	2013–2017	[52]
Switzerland	Switzerland	3.3% (51 out of 1540) ^3^	2013–2017	[52]
Germany	Germany	24.3% (1138 out of 4681) ^5,13^	2004–2009	[169]
	Germany	4.6% (534 out of 11,472) ^3^	2013–2017	[52]
	State of Hesse: Rhine-Main area	11.6% (81 out of 697) ^14^	2018–2020	[170]
Luxembourg	Luxembourg	3.3% (4 out of 122) ^3^	2013–2017	[52]
Belgium	Belgium	10.3% (6 out of 58) ^3^	2013–2017	[52]
The Netherlands	The Netherlands	8.4% (64 out of 761) ^3^	2013–2017	[52]
Denmark	Denmark	0.5% (2 out of 431) ^3^	2013–2017	[52]
Sweden	Sweden	2.6% (2 out of 77) ^3^	2013–2017	[52]
Norway	Norway	6.8% (5 out of 74) ^3^	2013–2017	[52]
Finland ^15^	Finland ^15^	9.6% (7 out of 73) ^3^	2013–2017	[52]
Italy	Southern Italy (Strait of Messina—narrow strait between Sicily and Calabria) ^15^	70.3% (175 out of 249) ^5,6^	2009	[171]
	Central and Northeastern Italy	2.3% (9 out of 385)	2005–2006	[93]
	Central Italy	1.7% (2 out of 117)	2012–2013	[172]
	Southern Italy (provinces in Campania region: Naples, Avellino, Salerno) ^15^	0.1% (2 out of 1311)	2015	[173]
	Italy	5% (46 out of 913) ^3^	2013–2017	[52]
	Northern Italy	29.1% (30 out of 103) ^2^	2003–2008	[174]
	Central Italy	4.6% (2 out of 43) ^2^	2003–2008	[174]
	Southern Italy	11.1% (2 out of 18) ^2^	2003–2008	[174]
France	Southern France	12.9% (18 out of 140)	2010–2012	[138]
	Most of samples from Northern France	63.2% (105 out of 166) ^6^	2006–2008	[6]
	France	9.1% (268 out of 2931) ^3^	2013–2017	[52]
Spain	Spain	3.6% (53 out of 1466) ^3^	2013–2017	[52]
United Kingdom	United Kingdom	1.7% (40 out of 2335) ^3^	2013–2017	[52]
Portugal	Portugal	0.7% (1 out of 143) ^3^	2013–2017	[52]
**Asia**				
Turkey	Eastern Turkey: Erzurum province ^15^	0.8% (1 out of 126)	2010–2012	[175]
Iran	Northwestern Iran: Meshkin Shahr County in Ardabil province ^15^	9.3% (4 out of 43)	2017–2018	[176]
China	Henan province: Zhengzhou city ^15^	5.4% (7 out of 130)	2017–2018	[177]
Japan	Japan ^15^	0.03% (1 out of 3463) ^3^	2013–2017	[52]
**North America**				
United States ^15^	United States (Canine Vector-Borne Disease Diagnostic Panel, Vector-Borne Disease Diagnostic Laboratory, North Carolina State University) ^15^	0.2% (18 out of 9367) ^3,16^	2015–2018	[5]

^1^ The results include prevalence of *B. canis* infection in dogs from regions endemic for western population of *D. reticulatus* and the European gap between western and eastern populations of *D. reticulatus*. ^2^ Only dogs with clinical signs suggesting babesiosis were included in the study. ^3^ Results of PCR-tested canine blood samples in a clinical laboratory (in some countries may include more cases with clinical signs suggestive of babesiosis). ^4^ Prevalence in free-ranging red foxes (*Vulpes vulpes*). ^5^ Seroprevalence. ^6^ Discrepancies between results presented in various parts of the article. ^7^ Unknown time of blood collection. ^8^ Blood collected from dogs infected with *Dirofilaria repens*. ^9^ Prevalence in free-ranging gray wolves (*Canis lupus*). ^10^ Prevalence in free-ranging golden jackals (*Canis aureus*). ^11^ Dogs were imported to Germany. ^12^ Two animals from other countries (one from Austria and one from Czech Republic). ^13^ Dogs imported from countries other than Germany, mainly from Portugal, Spain, Italy, Greece, and Turkey. ^14^ Only 14 out of 81 PCR products were sequenced; 13 sequences showed *B. canis* DNA, 1 sequence showed *B. vulpes* DNA. ^15^ Region/country out of range of *D. reticulatus* occurrence. ^16^ 9345 blood samples and 22 tissue samples.

**Table 5:** Prevalence of *Babesia vogeli* infection in *Rhipicephalus sanguineus* sensu lato ticks collected from vegetation/environment and dogs and other animals in various countries.

Country/Region		Prevalence of Infected Ticks (No. of Infections)	Time of Tick Collection	Ref.
**Ticks collected from vegetation/environment**				
Israel	Western Israel	2.3% (3 out of 131 pools) ^1^	2002–2003, 2007–2008	[220]
	Western Israel	0.8% (1 out of 124) ^2^	Before 2022 ^3^	[221]
Portugal	Southern Portugal: Faro District	0% (0 out of 230)	2012	[222]
**Ticks collected from dogs**				
**Western Pacific**				
United States	Western Pacific Ocean: Guam Island	1.3% (1 out of 75)	2010	[219]
**North America**				
Mexico	South-central Mexico: State of Morelos, Cuautla city	16.7% (3 out of 18)	Before 2017 ^3^	[218]
**South America**				
Brazil	Eastern Brazil: Pernambuco state (the municipality of Petrolina)	3% (3 out of 100)	2011–2012	[216]
	Midwestern Brazil: Mato Grosso State (Poconé municipality)	1.3% (5 out of 392)	2009	[215]
**Europe**				
Portugal	Southern Portugal: Faro District	0.3% (1 out of 321)	2012–2013	[222]
	Western Portugal: Lisbon and Setúbal districts	0% (0 out of 253)	2012–2013	[222]
	Northeastern Portugal: Guarda District	0% (0 out of 42)	2012–2013	[222]
Spain	Northeastern Spain (Barcelona metropolitan area)	3.2% (1 out of 31 pools) ^1,2,4,5^	2011–2013	[223]
France	Southern France	10.5% (26 out of 248) ^4^	2010–2012	[138]
Italy	Italy (78 provinces)	1.1% (10 out of 949 pools) ^1^	2016–2017	[224]
Ukraine	Southeastern Ukraine: Crimea (Sevastopol city)	0% (0 out of 52)	2016	[77]
Russia	Southern Russia: various cities (Astrakhan, Blagoveshchensk, Krasnodar, Sochi, Stavropol)	0% (0 out of 43)	2016	[77]
Chechnya	Central Chechnya: Grozny city	5.9% (1 out of 17)	2016	[77]
**Asia**				
Palestine	Palestine, 4 districts: Hebron, Jenin, Nablus, and Tubas	0.5% (1 out of 186)	2015	[225]
India	Southern India: Chennai city	2% (6 out of 294)	2018	[226]
Malaysia	Peninsular Malaysia	1.4% (2 out of 140)	Before 2018 ^3^	[227]
	Malaysia	33.3% (1 out of 3)	Before 2020 ^3^	[228]
Indonesia	Indonesia	0% (0 out of 78)	Before 2020 ^3^	[228]
Singapore	Singapore	0% (0 out of 4)	Before 2020 ^3^	[228]
Taiwan	Taiwan	0% (0 out of 21)	Before 2020 ^3^	[228]
	Northern and Western Taiwan	3.6% (21 out of 582)	2015–2017	[229]
Thailand	Thailand	0% (0 out of 34)	Before 2020 ^3^	[228]
Philippines	Philippines	0% (0 out of 90)	Before 2020 ^3^	[228]
	Northern Philippines: Metro Manila and Laguna	0.6% (1 out of 157)	Before 2018 ^3^	[230]
Vietnam	Vietnam	2.6% (3 out of 117)	Before 2020 ^3^	[228]
	Northern Vietnam: Hanoi and neighboring provinces	0.3% (1 out of 302)	2018	[231]
	Various provinces in southern, central, and northern Vietnam	3.6% (9 out of 251) ^4^	2010, 2018	[232]
China	China	0% (0 out of 20)	Before 2020 ^3^	[228]
	Eastern China: Jiangsu province (Taixing city)	3.4% (5 out of 146)	2012–2014	[233]
	Central China: Chongqing municipality	25% (4 out of 16)	Before 2012 ^3^	[213]
	Southeastern China: Guangdong province	3.6% (1 out of 28)	Before 2012 ^3^	[213]
	Southeastern China: Hainan province	3.3% (4 out of 121)	Before 2012 ^3^	[213]
	Southern China: Guangxi province	12.5% (11 out of 88)	Before 2012 ^3^	[213]
	Eastern China: Zhejiang province	6.7% (1 out of 15)	Before 2012 ^3^	[213]
**Australia**				
Australia	Australia: Queensland and Northern Territory	1.1% (2 out of 184) ^6^	2012–2015	[206]
**Africa**				
Egypt	Northern Egypt: Cairo and Giza cities	5.5% (8 out of 144)	Before 2022 ^3^	[234]
Tunisia	Tunisia, 4 locations: Zaga, Sidi Thabet, Somâa, and Bouhajla	0.6% (1 out of 160)	2006	[235]
Algeria	Northern Algeria: region of Djelfa and area of Bordj Bou Arreridj	13% (50 out of 384 pools) ^1^	2017–2019	[214]

^1^ Pooled ticks. ^2^ *R. turanicus*. ^3^ Unknown time of tick collection. ^4^ Ticks collected from dogs and other animals. ^5^ *B. vogeli* has been found in ticks collected from red foxes (*Vulpes vulpes*). ^6^ The only species in Australia previously considered as tropical lineage of *R. sanguineus* s.l. is classified now as *R. linnaei*.

**Table 6:** Prevalence of *Babesia vogeli* infection in dogs and in other canids.

Country/Region		Prevalence of Infected Dogs (No. of Infections)	Time of Blood Collection	Ref.
**Australia**				
Australia	Northern Australia: Tanami Desert, Kakadu National Park, Arnhem Land	21.4% (46 out of 215) ^1^	2000–2004	[207]
	Northern Australia: Arnhem Land: Maningrida	10% (13 out of 130) ^1^	2009–2010	[208]
	Northern Australia: Katherine city in the Northern Territory	8% (11 out of 138)	2009–2012	[209]
	Eastern Australia: southeastern Queensland	0% (0 out of 100)	2010	[208]
	Eastern Australia: southeastern Queensland	1% (1 out of 100)	2009–2012	[209]
	Central Australia: Ti-Tree communities in the Northern Territory	10% (5 out of 51) ^1^	2010	[246]
	Eastern Australia: communities in Moree, Mungindi, Toomelah, Boggabilla	4.4% (2 out of 45) ^1^	2013	[246]
	Various Aboriginal communities in western, central, northern, and eastern Australia	43.6% (17 out of 39) ^1^	2008–2009	[247]
	Australia	3.6% (27 out of 740) ^2^	2013–2017	[52]
**Asia**				
India	Southern India: Kerala State	6.3% (19 out of 300)	Before 2018 ^3^	[248]
	Southern India: Chennai city	10% (23 out of 230)	2018	[226]
	Southern India: Thrissur district of Kerala State	7.5% (6 out of 80) ^4^	Before 2017 ^3^	[249]
	Eight various states of India	1.2% (4 out of 330)	2012–2014	[250]
Nepal	Central Nepal: Kathmandu city	11.4% (8 out of 70) ^5^	2017	[251]
Pakistan	Eastern Pakistan: Bahawalpur city	0% (0 out of 49)	2018–2019	[252]
China	Southeastern China: Guangdong province (Shenzhen city)	11% (30 out of 272)	2018	[253]
	Eastern China: Jiangsu province (Taixing city)	11.3% (11 out of 97)	2012–2014	[233]
	Northern China: Gansu province	1.4% (2 out of 141)	2015–2016	[254]
	Northwestern China: Xinjiang Uygur Autonomous Region	16.7% (2 out of 12) ^6^	Before 2021 ^3^	[255]
	Southeastern China: Hong Kong	2.1% (34 out of 1648)	2018–2021	[256]
Iran	Northern Iran: Mazandaran province	0% (0 out of 75)	2018–2019	[252]
	Northern Iran: Teheran province	25% (10 out of 40) ^1^	2016–2017	[237]
	Western Iran: Provinces of Kermanshah, Khuzestan, and Hamadan	2% (4 out of 201)	2018–2019	[252]
	Central Iran: Yazd province	0% (0 out of 78)	2018–2019	[252]
Saudi Arabia	Central Saudi Arabia: Riyadh city	1.9% (1 out of 53)	2016	[257]
	Central Saudi Arabia: Riyadh province	0% (0 out of 74)	2018–2019	[258]
	Southwestern Saudi Arabia: Asir province	30% (21 out of 70) ^1^	2018–2019	[258]
Thailand	Northern Thailand: Mahasarakham province	6.3% (5 out of 79) ^1^	2014	[259]
	Central and western Thailand	18.2% (8 out of 44)	2020	[260]
	Western Thailand: Buriram province	2% (1 out of 49)	Before 2019 ^3^	[261]
	Southern Thailand: Pathum Thani province	0% (0 out of 95)	2022	[262]
	Southern Thailand	19.9% (28 out of 141) ^7^	2021	[263]
Japan	Southwestern Japan: Okinawa Island	6.2% (5 out of 80) ^1^	2001	[264]
	Japan	0.1% (5 out of 3463) ^2^	2013–2017	[52]
Singapore	Singapore	8.4% (118 out of 1396) ^2^	2013–2017	[52]
Taiwan	Various parts of Taiwan	9.5% (37 out of 388)	2015–2017	[245]
Malaysia	Peninsular Malaysia	2.1% (5 out of 240)	Before 2018 ^3^	[227]
Philippines	Northern Philippines: Metro Manila	5.3% (6 out of 114)	2013–2014	[265]
	Northern Philippines: Metro Manila and Laguna	6.8% (17 out of 248)	Before 2018 ^3^	[230]
Cambodia	Northern Cambodia: Preah Vihear province	32.7% (33 out of 101) ^1^	Before 2016 ^3^	[266]
Palestine	Palestine: 10 various districts	1.9% (7 out of 362)	2010, 2014, 2015	[225]
Turkey	Southeastern Turkey	1.4% (3 out of 219)	2015	[179]
**Africa**				
Egypt	Northern Egypt: governorates of Giza, Kafr El Sheikh, Qalyubia, and Gharbia	5.1% (14 out of 275)	2019	[267]
	Northern Egypt: Cairo	25.6% (62 out of 242)	Before 2021 ^3^	[268]
	Northern Egypt: Cairo and Giza cities	6.4% (8 out of 124)	Before 2022 ^3^	[234]
Sudan	Eastern Sudan: Barbar el Fugara village	2.6% (2 out of 78) ^1^	1997–2000	[49]
Tunisia	Four locations in various parts of Tunisia: Zaga, Sidi Thabet, Somâa, and Bouhajla	6.7% (12 out of 180)	2006	[235]
Nigeria	Central Nigeria: Jos South in Plateau State	1.2% (1 out of 84)	2011	[59]
	Central Nigeria: Abuja city	10.8% (52 out of 480) ^8^	2015–2016	[244]
Angola	Western Angola: Luanda city	5.8% (6 out of 103)	2013	[269]
Zambia	Southern Zambia: Lusaka, Mazabuka, Monze, and Shangombo cities	2.8% (7 out of 247)	2016	[63]
South Africa	South Africa: various provinces	4.4% (13 out of 297)	Before 2004 ^3^	[270]
	Northern South Africa: Northern Gauteng province and North West province	2.7% (14 out of 527)	2000–2006	[41]
	Central South Africa: Free State province	10.1% (13 out of 129)	2000–2006	[41]
**Europe**				
Albania	Central Albania: Tirana city	10% (3 out of 30)	2008	[161]
Romania	Romania	3.7% (4 out of 109) ^9^	2009–2010	[166]
	Southern Romania	2.7% (8 out of 300)	2017	[164]
	Eastern Romania: Iasi city	3.3% (3 out of 90) ^4^	2019	[40]
Hungary	Hungary	1.3% (1 out of 78) ^9^	2009–2010	[166]
Croatia	Croatia	0.2% (2 out of 848)	2007–2008	[157]
Serbia	Northern Serbia: Pančevo city	5.1% (3 out of 59)	2012–2014	[271]
	Southern Serbia: Niš and Prokuplje cities	0% (0 out of 66)	2012–2014	[271]
	Central Serbia: Belgrade city	0% (0 out of 111)	2015	[159]
Slovenia	Central Slovenia: Ljubljana city	1.3% (3 out of 238)	2000–2002	[160]
Austria	Austria	0.3% (3 out of 1146) ^2^	2013–2017	[52]
Switzerland	Switzerland	0.4% (7 out of 1540) ^2^	2013–2017	[52]
Italy	Northern Italy	1% (1 out of 103) ^4^	2003–2008	[174]
	Central Italy	16.3% (7 out of 43) ^4^	2003–2008	[174]
	Central Italy: Tuscany	2.6% (3 out of 117)	2012–2013	[172]
	Southern Italy	16.7% (3 out of 18) ^4^	2003–2008	[174]
	Southern Italy: provinces in Campania region	1.1% (15 out of 1311)	2015	[173]
	Italy	0.2% (2 out of 913) ^2^	2013–2017	[52]
Malta	Malta	4% (4 out of 99)	2013	[272]
France	Southern France	13.6% (19 out of 140)	2010–2012	[138]
	France	0.2% (7 out of 2931) ^2^	2013–2017	[52]
Spain	Central and southern Spain	1.2% (3 out of 250)	Before 2007 ^3^	[273]
	Spain	2.4% (36 out of 1466) ^2^	2013–2017	[52]
Portugal	Twelve various districts	35.7% (5 out of 14) ^10^	2017–2019	[236]
	Southern Portugal: Lisbon	2.8% (4 out of 142)	2016–2017	[274]
	Portugal	0.7% (1 out of 143) ^2^	2013–2017	[52]
The Netherlands	The Netherlands	0.8% (6 out of 761) ^2^	2013–2017	[52]
Belgium	Belgium	1.7% (1 out of 58) ^2^	2013–2017	[52]
Luxembourg	Luxembourg	0.8% (1 out of 122) ^2^	2013–2017	[52]
Germany	Germany	0.4% (49 out of 11,472) ^2^	2013–2017	[52]
Russia	Southwestern Russia: Rostov Oblast	4% (4 out of 100)	Before 2015 ^3^	[275]
United Kingdom	United Kingdom	0.3% (8 out of 2335) ^2^	2013–2017	[52]
**North America**				
Canada	Canada	0.2% (15 out of 6791) ^2^	2013–2017	[52]
United States	37 states of the United States and one Canadian province (Ontario)	1.5% (10 out of 673) ^2^	2000–2003	[276]
	Western United States: California	0.9% (4 out of 461) ^11,12^	2015–2019	[277]
	Western United States: California	7.1% (3 out of 42) ^4^	2009–2011	[278]
	Southern United States: Southern Texas	9% (11 out of 122)	2016	[279]
	United States	0.3% (29 out of 9367) ^2^	2015–2018	[5]
	United States	0.3% (194 out of 61,185) ^2^	2013–2017	[52]
Mexico	South-central Mexico: State of Morelos (Cuautla city)	10% (3 out of 30) ^4^	Before 2017 ^3^	[218]
El Salvador	Southern El Salvador: La Libertad and San Salvador departments	21% (21 out of 100) ^4^	2016–2017	[280]
Nicaragua	Southwestern Nicaragua: Rivas city	15.4% (6 out of 39)	2012	[281]
Turks and Caicos Islands	Turks and Caicos Islands	1.2% (1 out of 80) ^2^	2013–2017	[52]
Haiti	Haiti	7.7% (16 out of 207)	2013	[282]
Costa Rica	Northwestern Costa Rica	20% (8 out of 40)	2012	[283]
	Costa Rica	5.3% (24 out of 453) ^13^	2011–2014	[243]
	Costa Rica	31.2% (125 out of 400)	2011–2014	[243]
Saint Kitts and Nevis	Saint Kitts island	7.8% (14 out of 179)	2009–2011	[284]
	Saint Kitts and Nevis	3.6% (4 out of 110) ^2^	2013–2017	[52]
Grenada	School of Veterinary Medicine at St. George’s University	7% (5 out of 73)	2006	[285]
Trinidad and Tobago	Trinidad island	3.1% (10 out of 325)	2004–2006	[286]
**South America**				
Venezuela	Northern Venezuela: Falcón State	2.2% (3 out of 134)	Before 2007 ^3^	[273]
Colombia	Central Colombia: Bogotá, Villavicencio, and Bucaramanga cities	5.5% (5 out of 91)	Before 2012 ^3^	[287]
	Northern Colombia: Córdoba Department	26.2% (11 out of 42) ^4^	2013–2014	[288]
	Northern Colombia: Magdalena Department	13% (22 out of 169)	2017	[242]
	Colombia	1.8% (2 out of 113) ^2^	2013–2017	[52]
Ecuador	Eastern Pacific Ocean: Galápagos Islands (Isabela Island)	0% (0 out of 95) ^13^	2004	[289]
Brazil	Northern Brazil: Amazon region	10.6% (5 out of 47)	2008–2010	[290]
	Northeastern Brazil: the State of Paraíba (the municipality of Patos)	10% (10 out of 100) ^14^	2012	[241]
	Eastern Brazil: Pernambuco state (the municipality of Petrolina)	57.9% (234 out of 404) ^13^	2011–2012	[216]
	Eastern Brazil: Pernambuco state (Recife city)	4.8% (7 out of 146)	Before 2016 ^3^	[291]
	Eastern Brazil: Cerrado region	7.9% (5 out of 63)	2008–2010	[290]
	Southwestern Brazil: Mato Grosso do Sul State (Corumbá municipality)	14.3% (6 out of 42)	2013–2015	[292]
	Midwestern Brazil: Mato Grosso State (Poconé municipality)	3.1% (10 out of 320)	2009	[215]
	Southeastern Brazil: Minas Gerais State (regions: Lavras, Belo Horizonte, Nanuque)	28.7% (70 out of 244) ^13^	2004	[293]
	Southeastern Brazil: Minas Gerais State (regions: Lavras, Belo Horizonte, Nanuque)	17.1% (12 out of 70) ^15^	2004	[293]
	Southeastern Brazil: Minas Gerais State (regions: Lavras, Belo Horizonte, Nanuque)	9.9% (25 out of 252) ^16^	2004	[240]
	Southeastern Brazil: Minas Gerais State (regions: Lavras, Belo Horizonte, Nanuque)	10.8% (18 out of 166) ^17^	2004–2005	[240]
	Southeastern Brazil: Rio de Janeiro State (Itaguaí municipality)	14.1% (55 out of 390) ^5^	Before 2018 ^3^	[239]
	Southeastern Brazil: Espírito Santo State (Alegre, Colatina, Santa Teresa, Serra, Vila Velha, Vitória minicipalities)	1.3% (5 out of 378)	Before 2018 ^3^	[294]
	Southern Brazil: Paraná State	11% (20 out of 182)	2014	[238]
	Brazil	1.5% (17 out of 1105) ^2^	2013–2017	[52]
Paraguay	Southwestern Paraguay: Asunción city	5.5% (21 out of 384)	2015–2016	[295]
	Paraguay	9.2% (37 out of 400) ^2^	2013–2017	[52]
Peru	Northwestern Peru: Piura city	1.4% (3 out of 212)	2014–2015	[296]
Argentina	Central Argentina: Córdoba and Santa Fé provinces	7.7% (5 out of 65)	Before 2016 ^3^	[297]
	Southern Argentina: Santa Cruz province	0% (0 out of 48) ^18^	2010–2015	[298]
Chile	Middle Chile: Coquimbo region	7.9% (5 out of 63)	2018–2019	[299]

^1^ Free-roaming dogs. ^2^ Results of PCR-tested canine blood samples in a clinical laboratory (in some countries may include more cases with clinical signs suggestive of babesiosis). ^3^ Unknown time of tick collection. ^4^ Dogs with clinical signs suggesting babesiosis or similar disease. ^5^ Discrepancies between results in various parts of the article. ^6^ Prevalence in red foxes (*Vulpes vulpes*). ^7^ Dogs in a shelter. ^8^ Prevalence based on microscopic examination of blood smears, infection with *B. vogeli* confirmed by PCR and sequencing (unknown primer sequences and PCR details). ^9^ Dogs were imported to Germany. ^10^ Only dogs infected with *Rickettsia massiliae* were examined for the presence of *Babesia* spp. ^11^ Splenic samples from coyotes (*Canis latrans*) were examined. ^12^ Total of 4 out of 14 sequenced PCR products were 100% homologous with *B. vogeli*. ^13^ Seroprevalence. ^14^ Dogs with tick infestation. ^15^ PCR testing only of 70 seropositive dogs out of 244 examined animals. ^16^ Prevalence in dry season April-September 2004. ^17^ Prevalence in rainy season October 2004 to March 2005. ^18^ Prevalence in South American gray foxes (*Lycalopex griseus*).

## Data Availability

Not applicable.

## References

[1] Baneth G., Florin-Christensen M., Schnittger L. (2018). *Babesia* of Domestic Dogs. Parasitic Protozoa of Farm Animals and Pets.

[2] Baneth G., Nachum-Biala Y., Birkenheuer A.J., Schreeg M.E., Prince H., Florin-Christensen M., Schnittger L., Aroch I. (2020). A new piroplasmid species infecting dogs: Morphological and molecular characterization and pathogeny of *Babesia negevi* n. sp.. Parasit. Vectors.

[3] Köster L.S., Lobetti R.G., Kelly P. (2015). Canine babesiosis: A perspective on clinical complications, biomarkers, and treatment. Vet. Med. Res. Rep..

[4] Shaw M., Kolba N., Huffman J.E. (2015). *Babesia* spp. in *Ursus americanus* (Black Bear) in New Jersey. Northeast. Nat..

[5] Barash N.R., Thomas B., Birkenheuer A.J., Breitschwerdt E.B., Lemler E., Qurollo B.A. (2019). Prevalence of *Babesia* spp. and clinical characteristics of *Babesia vulpes* infections in North American dogs. J. Vet. Intern. Med..

[6] Fritz D. (2010). A PCR study of piroplasms in 166 dogs and 111 horses in France (March 2006 to March 2008). Parasitol. Res..

[7] Hornok S., Horváth G., Takács N., Kontschán J., Szőke K., Farkas R. (2018). Molecular identification of badger-associated *Babesia* sp. DNA in dogs: Updated phylogeny of piroplasms infecting Caniformia. Parasit. Vectors.

[8] Birkenheuer A.J., Neel J., Ruslander D., Levy M.G., Breitschwerdt E.B. (2004). Detection and molecular characterization of a novel large *Babesia* species in a dog. Vet. Parasitol..

[9] Holman P.J., Backlund B.B., Wilcox A.L., Stone R., Stricklin A.L., Bardin K.E. (2009). Detection of a large unnamed *Babesia* piroplasm originally identified in dogs in North Carolina in a dog with no history of travel to that state. J. Am. Vet. Med. Assoc..

[10] Sikorski L.E., Birkenheuer A.J., Holowaychuk M.K., McCleary-Wheeler A.L., Davis J.M., Littman M.P. (2010). Babesiosis Caused by a Large *Babesia* Species in 7 Immunocompromised Dogs. J. Vet. Intern. Med..

[11] Marks Stowe D.A., Birkenheuer A.J., Grindem C.B. (2012). Pathology in practice. Intraerythrocytic infection with organisms consistent with a large *Babesia* sp.. J. Am. Vet. Med. Assoc..

[12] Jacobson L.S. (2006). The South African form of severe and complicated canine babesiosis: Clinical advances 1994–2004. Vet. Parasitol..

[13] Chauvin A., Moreau E., Bonnet S., Plantard O., Malandrin L. (2009). Babesia and its hosts: Adaptation to long-lasting interactions as a way to achieve efficient transmission. Vet. Res..

[14] Djokic V., Rocha S.C., Parveen N. (2021). Lessons Learned for Pathogenesis, Immunology, and Disease of Erythrocytic Parasites: Plasmodium and Babesia. Front. Cell. Infect. Microbiol..

[15] Leisewitz A., Goddard A., De Gier J., Van Engelshoven J., Clift S., Thompson P., Schoeman J.P. (2019). Disease severity and blood cytokine concentrations in dogs with natural Babesia rossi infection. Parasite Immunol..

[16] Gójska-Zygner O., Bartosik J., Górski P., Zygner W. (2019). Hyponatraemia and syndrome of inappropriate antidiuretic hormone secretion in non-azotaemic dogs with babesiosis associated with decreased arterial blood pressure. J. Vet. Res..

[17] Zygner W., Wędrychowicz H. (2009). Influence of anaemia on azotaemia in dogs infected with *Babesia canis* in Poland. Bull. Vet. Inst. Pulawy.

[18] Rafaj R.B., Matijatko V., Kis I., Kučer N., Živičnjak T., Lemo N., Žvorc Z., Brkljačić M., Mrljak V. (2009). Alterations in some blood coagulation parameters in naturally occurring cases of canine babesiosis. Acta Vet. Hung..

[19] Gójska-Zygner O., Zygner W. (2015). Hyperaldosteronism and its association with hypotension and azotaemia in canine babesiosis. Vet. Q..

[20] Schoeman J.P., Rees P., Herrtage M.E. (2007). Endocrine predictors of mortality in canine babesiosis caused by *Babesia canis rossi*. Vet. Parasitol..

[21] Matijatko V., Kiš I., Torti M., Brkljačić M., Kučer N., Rafaj R.B., Grden D., Živičnjak T., Mrljak V. (2009). Septic shock in canine babesiosis. Vet. Parasitol..

[22] Kuleš J., de Torre-Minguela C., Barić Rafaj R., Gotić J., Nižić P., Ceron J.J., Mrljak V. (2016). Plasma biomarkers of SIRS and MODS associated with canine babesiosis. Res. Vet. Sci..

[23] Kuleš J., Bilić P., Beer Ljubić B., Gotić J., Crnogaj M., Brkljačić M., Mrljak V. (2018). Glomerular and tubular kidney damage markers in canine babesiosis caused by *Babesia canis*. Ticks Tick-Borne Dis..

[24] Zygner W., Rodo A., Gójska-Zygner O., Górski P., Bartosik J., Kotomski G. (2021). Disorders in blood circulation as a probable cause of death in dogs infected with *Babesia canis*. J. Vet. Res..

[25] Lobetti R.G., Jacobson L.S. (2001). Renal involvement in dogs with babesiosis. J. S. Afr. Vet. Assoc..

[26] Möhr A.J., Lobetti R.G., van der Lugt J.J. (2000). Acute pancreatitis: A newly recognised potential complication of canine babesiosis. J. S. Afr. Vet. Assoc..

[27] Dvir E., Lobetti R.G., Jacobson L.S., Pearson J., Becker P.J. (2004). Electrocardiographic changes and cardiac pathology in canine babesiosis. J. Vet. Cardiol..

[28] Zygner W., Gójska-Zygner O., Norbury L.J. (2023). Pathogenesis of Anemia in Canine Babesiosis: Possible Contribution of Pro-Inflammatory Cytokines and Chemokines—A Review. Pathogens.

[29] Penzhorn B.L., Harrison-White R.F., Stoltsz W.H. (2020). Completing the cycle: *Haemaphysalis elliptica*, the vector of *Babesia rossi*, is the most prevalent tick infesting black-backed jackals (*Canis mesomelas*), an indigenous reservoir host of *B. rossi* in South Africa. Ticks Tick-Borne Dis..

[30] Kamani J. (2021). Molecular evidence indicts *Haemaphysalis leachi* (Acari: Ixodidae) as the vector of *Babesia rossi* in dogs in Nigeria, West Africa. Ticks Tick-Borne Dis..

[31] Kamani J., Chung P.J., Lee C.C., Chung Y.T. (2018). In search of the vector(s) of *Babesia rossi* in Nigeria: Molecular detection of *B. rossi* DNA in *Rhipicephalus sanguineus* sensu lato (Acari: Ixodidae) ticks collected from dogs, circumstantial evidence worth exploring. Exp. Appl. Acarol..

[32] Orkun Ö., Karaer Z., Çakmak A., Nalbantoğlu S. (2014). Identification of Tick-Borne Pathogens in Ticks Feeding on Humans in Turkey. PLoS Negl. Trop. Dis..

[33] Orkun Ö., Karaer Z. (2017). Molecular characterization of *Babesia* species in wild animals and their ticks in Turkey. Infect. Genet. Evol..

[34] Penzhorn B.L. (2011). Why is Southern African canine babesiosis so virulent? An evolutionary perspective. Parasit. Vectors.

[35] Penzhorn B.L., Vorster I., Harrison-White R.F., Oosthuizen M.C. (2017). Black-backed jackals (*Canis mesomelas*) are natural hosts of *Babesia rossi*, the virulent causative agent of canine babesiosis in sub-Saharan Africa. Parasit. Vectors.

[36] Viljoen S., O’Riain M.J., Penzhorn B.L., Drouilly M., Vorster I., Bishop J.M. (2021). Black-backed jackals (*Canis mesomelas*) from semi-arid rangelands in South Africa harbour *Hepatozoon canis* and a *Theileria* species but apparently not *Babesia rossi*. Vet. Parasitol. Reg. Stud. Rep..

[37] Matjila P.T., Leisewitz A.L., Jongejan F., Bertschinger H.J., Penzhorn B.L. (2008). Molecular detection of *Babesia rossi* and *Hepatozoon* sp. in African wild dogs (*Lycaon pictus*) in South Africa. Vet. Parasitol..

[38] Shabangu N., Penzhorn B.L., Oosthuizen M.C., Vorster I., van Schalkwyk O.L., Harrison-White R.F., Matjila P.T. (2021). A shared pathogen: *Babesia rossi* in domestic dogs, black-backed jackals (*Canis mesomelas*) and African wild dogs (*Lycaon pictus*) in South Africa. Vet. Parasitol..

[39] Prager K.C., Mazet J.A.K., Munson L., Cleaveland S., Donnelly C.A., Dubovi E.J., Szykman Gunther M., Lines R., Mills G., Davies-Mostert H.T. (2012). The effect of protected areas on pathogen exposure in endangered African wild dog (*Lycaon pictus*) populations. Biol. Conserv..

[40] Ciuca L., Martinescu G., Miron L.D., Roman C., Acatrinei D., Cringoli G., Rinaldi L., Maurelli M.P. (2021). Occurrence of *Babesia* Species and Co-Infection with *Hepatozoon canis* in Symptomatic Dogs and in Their Ticks in Eastern Romania. Pathogens.

[41] Matjila P.T., Leisewitz A.L., Jongejan F., Penzhorn B.L. (2008). Molecular detection of tick-borne protozoal and ehrlichial infections in domestic dogs in South Africa. Vet. Parasitol..

[42] Adamu M., Troskie M., Oshadu D.O., Malatji D.P., Penzhorn B.L., Matjila P.T. (2014). Occurrence of tick-transmitted pathogens in dogs in Jos, Plateau State, Nigeria. Parasit. Vectors.

[43] Sasaki M., Omobowale O., Tozuka M., Ohta K., Matsuu A., Nottidge H.O., Hirata H., Ikadai H., Oyamada T. (2007). Molecular survey of *Babesia canis* in dogs in Nigeria. J. Vet. Med. Sci..

[44] Hirata H., Omobowale T., Adebayo O., Asanuma N., Haraguchi A., Murakami Y., Kusakisako K., Ikeda K., Asakawa M., Suzuki K. (2022). Identification and phylogenetic analysis of *Babesia* parasites in domestic dogs in Nigeria. J. Vet. Med. Sci..

[45] Proboste T., Kalema-Zikusoka G., Altet L., Solano-Gallego L., Fernández de Mera I.G., Chirife A.D., Muro J., Bach E., Piazza A., Cevidanes A. (2015). Infection and exposure to vector-borne pathogens in rural dogs and their ticks, Uganda. Parasit. Vectors.

[46] Chatanga E., Kainga H., Razemba T., Ssuna R., Swennen L., Hayashida K., Sugimoto C., Katakura K., Nonaka N., Nakao R. (2021). Molecular detection and characterization of tick-borne hemoparasites and Anaplasmataceae in dogs in major cities of Malawi. Parasitol. Res..

[47] Ngoka I.T., Mbogo K., Kyallo M., Oduori D.O., Pelle R. (2021). Genetic detection and phylogenetic relationship of *Babesia* species infecting domestic dogs from select regions in Kenya. Sci. Afr..

[48] Sili G., Byaruhanga C., Horak I., Steyn H., Chaisi M., Oosthuizen M.C., Neves L. (2021). Ticks and tick-borne pathogens infecting livestock and dogs in Tchicala-Tcholoanga, Huambo Province, Angola. Parasitol. Res..

[49] Oyamada M., Davoust B., Boni M., Dereure J., Bucheton B., Hammad A., Itamoto K., Okuda M., Inokuma H. (2005). Detection of *Babesia canis rossi*, *B. canis vogeli*, and *Hepatozoon canis* in Dogs in a Village of Eastern Sudan by Using a Screening PCR and Sequencing Methodologies. Clin. Diagn. Lab. Immunol..

[50] Nalubamba K.S., Mudenda N.B., Namwila M.M., Mulenga C.S., Bwalya E.C., M’kandawire E., Saasa N., Hankanga C., Oparaocha E., Simuunza M. (2015). A Study of Naturally Acquired Canine Babesiosis Caused by Single and Mixed *Babesia* Species in Zambia: Clinicopathological Findings and Case Management. J. Parasitol. Res..

[51] Allison R.W., Yeagley T.J., Levis K., Reichard M.V. (2011). *Babesia canis rossi* infection in a Texas dog. Vet. Clin. Pathol..

[52] Birkenheuer A.J., Buch J., Beall M.J., Braff J., Chandrashekar R. (2020). Global distribution of canine *Babesia* species identified by a commercial diagnostic laboratory. Vet. Parasitol. Reg. Stud. Rep..

[53] Egorov D.S., Balandina V.N., Kruchkova E.N., Kuzmichev V.V., Egorov S.V. *Babesia* spp. infections of dogs in the Upper-Volga Area. Proceedings of the 16th Scientific Conference on the “Theory and Practice of the Struggle against Parasitic Diseases”.

[54] Allan R.E. (2016). The Occurrence of Tick-Borne Pathogens, in Dogs in Welfare Organisations and Townships of Cape Town. Master’s Thesis.

[55] Mofokeng L.S., Taioe O.M., Smit N.J., Thekisoe O.M.M. (2020). Parasites of veterinary importance from domestic animals in uMkhanyakude district of KwaZulu-Natal province. J. S. Afr. Vet. Assoc..

[56] Mellanby R.J., Handel I.G., Clements D.N., Bronsvoort B.M., Lengeling A., Schoeman J.P. (2011). Breed and Sex Risk Factors for Canine Babesiosis in South Africa. J. Vet. Intern. Med..

[57] Morters M.K., Archer J., Ma D., Matthee O., Goddard A., Leisewitz A.L., Matjila P.T., Wood J.L.N., Schoeman J.P. (2020). Long-term follow-up of owned, free-roaming dogs in South Africa naturally exposed to *Babesia rossi*. Int. J. Parasitol..

[58] Golezardy H. (2011). Prevalence of *Babesia* Species and Associated Ticks (Acari: Ixodidae) in Captive Cheetah (*Acinonyx jubatus*) Populations in South Africa. Ph.D. Thesis.

[59] Kamani J., Baneth G., Mumcuoglu K.Y., Waziri N.E., Eyal O., Guthmann Y., Harrus S. (2013). Molecular Detection and Characterization of Tick-borne Pathogens in Dogs and Ticks from Nigeria. PLoS Negl. Trop. Dis..

[60] Takeet M.I., Oyewusi A.J., Abakpa S.A., Daramola O.O., Peters S.O. (2017). Genetic diversity among *Babesia rossi* detected in naturally infected dogs in Abeokuta, Nigeria, based on 18S rRNA gene sequences. Acta Parasitol..

[61] Gruenberger I., Liebich A.V., Ajibade T.O., Obebe O.O., Ogbonna N.F., Wortha L.N., Unterköfler M.S., Fuehrer H.P., Ayinmode A.B. (2023). Vector-Borne Pathogens in Guard Dogs in Ibadan, Nigeria. Pathogens.

[62] Williams B.M., Berentsen A., Shock B.C., Teixiera M., Dunbar M.R., Becker M.S., Yabsley M.J. (2014). Prevalence and diversity of *Babesia*, *Hepatozoon*, *Ehrlichia*, and *Bartonella* in wild and domestic carnivores from Zambia, Africa. Parasitol. Res..

[63] Qiu Y., Kaneko C., Kajihara M., Ngonda S., Simulundu E., Muleya W., Thu M.J., Hang’ombe M.B., Katakura K., Takada A. (2018). Tick-borne haemoparasites and Anaplasmataceae in domestic dogs in Zambia. Ticks Tick Borne Dis..

[64] Van Wyk C.L., Mtshali K., Taioe M.O., Terera S., Bakkes D., Ramatla T., Xuan X., Thekisoe O. (2022). Detection of Ticks and Tick-Borne Pathogens of Urban Stray Dogs in South Africa. Pathogens.

[65] Lewis B.D., Penzhorn B.L., Lopez-Rebollar L.M., De Waal D.T. (1996). Isolation of a South African vector-specific strain of *Babesia canis*. Vet. Parasitol..

[66] Horak I.G. (1995). Ixodid ticks collected at the faculty of veterinary science, Onderstepoort, from dogs diagnosed with *Babesia canis* infection. J. S. Afr. Vet. Assoc..

[67] Apanaskevich D.A., Horak I.G., Camicas J.L. (2007). Redescription of *Haemaphysalis* (*Rhipistoma*) *elliptica* (Koch, 1844), an old taxon of the *Haemaphysalis* (*Rhipistoma*) *leachi* group from East and southern Africa, and of *Haemaphysalis* (*Rhipistoma*) *leachi* (Audouin, 1826) (Ixodida, Ixodidae). Onderstepoort J. Vet. Res..

[68] Uilenberg G., Franssen F.F., Perié N.M., Spanjer A.A. (1989). Three groups of *Babesia canis* distinguished and a proposal for nomenclature. Vet. Q..

[69] Nalubamba K.S., Hankanga C., Mudenda N.B., Masuku M. (2011). The epidemiology of canine *Babesia* infections in Zambia. Prev. Vet. Med..

[70] Bajer A., Beck A., Beck R., Behnke J.M., Dwużnik-Szarek D., Eichenberger R.M., Farkas R., Fuehrer H.P., Heddergott M., Jokelainen P. (2022). Babesiosis in Southeastern, Central and Northeastern Europe: An Emerging and Re-Emerging Tick-Borne Disease of Humans and Animals. Microorganisms.

[71] Karbowiak G. (2014). The occurrence of the *Dermacentor reticulatus* tick—Its expansion to new areas and possible causes. Ann. Parasitol..

[72] Kjær L.J., Soleng A., Edgar K.S., Lindstedt H.E.H., Paulsen K.M., Andreassen Å.K., Korslund L., Kjelland V., Slettan A., Stuen S. (2019). A large-scale screening for the taiga tick, *Ixodes persulcatus*, and the meadow tick, *Dermacentor reticulatus*, in southern Scandinavia, 2016. Parasit. Vectors.

[73] Földvári G., Široký P., Szekeres S., Majoros G., Sprong H. (2016). *Dermacentor reticulatus*: A vector on the rise. Parasit. Vectors.

[74] Rubel F., Brugger K., Pfeffer M., Chitimia-Dobler L., Didyk Y.M., Leverenz S., Dautel H., Kahl O. (2016). Geographical distribution of *Dermacentor marginatus* and *Dermacentor reticulatus* in Europe. Ticks Tick Borne Dis..

[75] Olivieri E., Zanzani S.A., Latrofa M.S., Lia R.P., Dantas-Torres F., Otranto D., Manfredi M.T. (2016). The southernmost foci of *Dermacentor reticulatus* in Italy and associated *Babesia canis* infection in dogs. Parasit. Vectors.

[76] Rubel F., Brugger K., Belova O.A., Kholodilov I.S., Didyk Y.M., Kurzrock L., García-Pérez A.L., Kahl O. (2020). Vectors of disease at the northern distribution limit of the genus *Dermacentor* in Eurasia: *D. reticulatus* and *D. silvarum*. Exp. Appl. Acarol..

[77] Livanova N.N., Fomenko N.V., Akimov I.A., Ivanov M.J., Tikunova N.V., Armstrong R., Konyaev S.V. (2018). Dog survey in Russian veterinary hospitals: Tick identification and molecular detection of tick-borne pathogens. Parasit. Vectors.

[78] Dautel H., Dippel C., Oehme R., Hartelt K., Schettler E. (2006). Evidence for an increased geographical distribution of *Dermacentor reticulatus* in Germany and detection of *Rickettsia* sp. RpA4. Int. J. Med. Microbiol..

[79] Zygner W., Górski P., Wędrychowicz H. (2009). New localities of *Dermacentor reticulatus* tick (vector of *Babesia canis canis*) in central and eastern Poland. Pol. J. Vet. Sci..

[80] Kiewra D., Czulowska A. (2013). Evidence for an increased distribution range of *Dermacentor reticulatus* in south-west Poland. Exp. Appl. Acarol..

[81] Drehmann M., Springer A., Lindau A., Fachet K., Mai S., Thoma D., Schneider C.R., Chitimia-Dobler L., Bröker M., Dobler G. (2020). The Spatial Distribution of *Dermacentor* Ticks (Ixodidae) in Germany—Evidence of a Continuing Spread of *Dermacentor reticulatus*. Front. Vet. Sci..

[82] Dwużnik-Szarek D., Mierzejewska E.J., Rodo A., Goździk K., Behnke-Borowczyk J., Kiewra D., Kartawik N., Bajer A. (2021). Monitoring the expansion of *Dermacentor reticulatus* and occurrence of canine babesiosis in Poland in 2016–2018. Parasit. Vectors.

[83] Noll M., Wall R., Makepeace B.L., Vineer H.R. (2023). Distribution of ticks in the Western Palearctic: An updated systematic review (2015–2021). Parasit. Vectors.

[84] Mierzejewska E.J., Estrada-Peña A., Bajer A. (2017). Spread of *Dermacentor reticulatus* is associated with the loss of forest area. Exp. Appl. Acarol..

[85] Hornok S., Farkas R. (2009). Influence of biotope on the distribution and peak activity of questing ixodid ticks in Hungary. Med. Vet. Entomol..

[86] García-Sanmartín J., Barandika J.F., Juste R.A., García-Pérez A.L., Hurtado A. (2008). Distribution and molecular detection of *Theileria* and *Babesia* in questing ticks from northern Spain. Med. Vet. Entomol..

[87] Cieniuch S., Stańczak J., Ruczaj A. (2009). The first detection of *Babesia* EU1 and *Babesia canis canis* in *Ixodes ricinus* ticks (Acari, Ixodidae) collected in urban and rural areas in northern Poland. Pol. J. Microbiol..

[88] Solano-Gallego L., Sainz Á., Roura X., Estrada-Peña A., Miró G. (2016). A review of canine babesiosis: The European perspective. Parasit. Vectors.

[89] Capligina V., Seleznova M., Akopjana S., Freimane L., Lazovska M., Krumins R., Kivrane A., Namina A., Aleinikova D., Kimsis J. (2020). Large-scale countrywide screening for tick-borne pathogens in field-collected ticks in Latvia during 2017–2019. Parasit. Vectors.

[90] Liberska J., Michalik J., Pers-Kamczyc E., Wierzbicka A., Lane R.S., Rączka G., Opalińska P., Skorupski M., Dabert M. (2021). Prevalence of *Babesia canis* DNA in *Ixodes ricinus* ticks collected in forest and urban ecosystems in west-central Poland. Ticks Tick Borne Dis..

[91] Estrada-Peña A., Roura X., Sainz A., Miró G., Solano-Gallego L. (2017). Species of ticks and carried pathogens in owned dogs in Spain: Results of a one-year national survey. Ticks Tick Borne Dis..

[92] Trotta M., Nicetto M., Fogliazza A., Montarsi F., Caldin M., Furlanello T., Solano-Gallego L. (2012). Detection of *Leishmania infantum*, *Babesia canis*, and rickettsiae in ticks removed from dogs living in Italy. Ticks Tick Borne Dis..

[93] Cassini R., Zanutto S., Frangipane di Regalbono A., Gabrielli S., Calderini P., Moretti A., Tampieri M.P., Pietrobelli M. (2009). Canine piroplasmosis in Italy: Epidemiological aspects in vertebrate and invertebrate hosts. Vet. Parasitol..

[94] Silaghi C., Woll D., Hamel D., Pfister K., Mahling M., Pfeffer M. (2012). *Babesia* spp. and *Anaplasma phagocytophilum* in questing ticks, ticks parasitizing rodents and the parasitized rodents—Analyzing the host-pathogen-vector interface in a metropolitan area. Parasit. Vectors.

[95] Schaarschmidt D., Gilli U., Gottstein B., Marreros N., Kuhnert P., Daeppen J.A., Rosenberg G., Hirt D., Frey C.F. (2013). Questing *Dermacentor reticulatus* harbouring *Babesia canis* DNA associated with outbreaks of canine babesiosis in the Swiss Midlands. Ticks Tick Borne Dis..

[96] Dzięgiel B., Kubrak T., Adaszek Ł., Dębiak P., Wyłupek D., Bogucka-Kocka A., Lechowski J., Winiarczyk S. (2014). Prevalence of *Babesia canis*, *Borrelia burgdorferi* sensu lato, and *Anaplasma phagocytophilum* in hard ticks collected from meadows of Lubelskie Voivodship (eastern Poland). Bull. Vet. Inst. Pulawy.

[97] Wójcik-Fatla A., Zając V., Sawczyn A., Cisak E., Dutkiewicz J. (2015). *Babesia* spp. in questing ticks from eastern Poland: Prevalence and species diversity. Parasitol. Res..

[98] Zając V., Wójcik-Fatla A., Sawczyn A., Cisak E., Sroka J., Kloc A., Zając Z., Buczek A., Dutkiewicz J., Bartosik K. (2017). Prevalence of infections and co-infections with 6 pathogens in *Dermacentor reticulatus* ticks collected in eastern Poland. Ann. Agric. Environ. Med..

[99] Bilbija B., Spitzweg C., Papoušek I., Fritz U., Földvári G., Mullett M., Ihlow F., Sprong H., Civáňová Křížová K., Anisimov N. (2023). *Dermacentor reticulatus*—A tick on its way from glacial refugia to a panmictic Eurasian population. Int. J. Parasitol..

[100] Kloch A., Mierzejewska E.J., Karbowiak G., Slivinska K., Alsarraf M., Rodo A., Kowalec M., Dwużnik D., Didyk Y.M., Bajer A. (2017). Origins of recently emerged foci of the tick *Dermacentor reticulatus* in central Europe inferred from molecular markers. Vet. Parasitol..

[101] Dwużnik-Szarek D., Mierzejewska E.J., Kiewra D., Czułowska A., Robak A., Bajer A. (2022). Update on prevalence of *Babesia canis* and *Rickettsia* spp. in adult and juvenile *Dermacentor reticulatus* ticks in the area of Poland (2016–2018). Sci. Rep..

[102] Mierzejewska E.J., Pawełczyk A., Radkowski M., Welc-Falęciak R., Bajer A. (2015). Pathogens vectored by the tick, *Dermacentor reticulatus*, in endemic regions and zones of expansion in Poland. Parasit. Vectors.

[103] Król N., Kiewra D., Lonc E., Janaczyk B., Chodorowska-Skubiszewska A., Dzięcioł M., Gola M., Gruszka R., Jackowska-Szlachcic E., Jagiełło M. (2016). *Dermacentor reticulatus* (Fabricius, 1794) and *Babesia canis* (Piana et Galli-Valerio, 1895) as the parasites of companion animals (dogs and cats) in the Wrocław area, south-western Poland. Ann. Parasitol..

[104] Grochowska A., Dunaj-Małyszko J., Pancewicz S., Czupryna P., Milewski R., Majewski P., Moniuszko-Malinowska A. (2022). Prevalence of Tick-Borne Pathogens in Questing *Ixodes ricinus* and *Dermacentor reticulatus* Ticks Collected from Recreational Areas in Northeastern Poland with Analysis of Environmental Factors. Pathogens.

[105] Grochowska A., Dunaj J., Pancewicz S., Czupryna P., Majewski P., Wondim M., Tryniszewska E., Moniuszko-Malinowska A. (2021). Detection of *Borrelia burgdorferi* s.l., *Anaplasma phagocytophilum* and *Babesia* spp. in *Dermacentor reticulatus* ticks found within the city of Białystok, Poland—First data. Exp. Appl. Acarol..

[106] Dunaj J., Trzeszczkowski A., Moniuszko-Malinowska A., Rutkowski K., Pancewicz S. (2021). Assessment of tick-borne pathogens presence in *Dermacentor reticulatus* ticks in north-eastern Poland. Adv. Med. Sci..

[107] Silaghi C., Weis L., Pfister K. (2020). *Dermacentor reticulatus* and *Babesia canis* in Bavaria (Germany)—A Georeferenced Field Study with Digital Habitat Characterization. Pathogens.

[108] Beelitz P., Schumacher S., Marholdt F., Pfister K., Silaghi C. (2012). The prevalence of *Babesia canis canis* in marsh ticks (*Dermacentor reticulatus*) in the Saarland. Berl. Munch. Tierarztl. Wochenschr..

[109] Leschnik M.W., Khanakah G., Duscher G., Wille-Piazzai W., Hörweg C., Joachim A., Stanek G. (2012). Species, developmental stage and infection with microbial pathogens of engorged ticks removed from dogs and questing ticks. Med. Vet. Entomol..

[110] Rybarova M., Honsova M., Papousek I., Siroky P. (2017). Variability of species of *Babesia* Starcovici, 1893 in three sympatric ticks (*Ixodes ricinus*, *Dermacentor reticulatus* and *Haemaphysalis concinna*) at the edge of Pannonia in the Czech Republic and Slovakia. Folia Parasitol..

[111] Duh D., Slovák M., Saksida A., Strašek K., Petrovec M., Avšič-Županc T. (2006). Molecular detection of *Babesia canis* in *Dermacentor reticulatus* ticks collected in Slovakia. Biologia.

[112] Svehlová A., Berthová L., Sallay B., Boldiš V., Sparagano O.A., Spitalská E. (2014). Sympatric occurrence of *Ixodes ricinus*, *Dermacentor reticulatus* and *Haemaphysalis concinna* ticks and *Rickettsia* and *Babesia* species in Slovakia. Ticks Tick Borne Dis..

[113] Řeháčková K., Haláková M., Víchová B., Kočišová A. (2016). Epizootiological Study of the Occurrence of Canine Babesiosis in Southwestern Slovakia. Folia Vet..

[114] Kubelová M., Tkadlec E., Bednář M., Roubalová E., Siroký P. (2011). West-to-east differences of *Babesia canis canis* prevalence in *Dermacentor reticulatus* ticks in Slovakia. Vet. Parasitol..

[115] Majláthová V., Majláth I., Víchová B., Gul’ová I., Derdáková M., Sesztáková E., Pet’ko B. (2011). Polymerase chain reaction Confirmation of *Babesia canis canis* and *Anaplasma phagocytophilum* in Dogs Suspected of Babesiosis in Slovakia. Vector Borne Zoonotic Dis..

[116] Sands B., Lihou K., Lait P., Wall R. (2022). Prevalence of *Babesia* spp. pathogens in the ticks *Dermacentor reticulatus* and *Ixodes ricinus* in the UK. Acta Trop..

[117] de Marco M.D.M.F., Hernández-Triana L.M., Phipps L.P., Hansford K., Mitchell E.S., Cull B., Swainsbury C.S., Fooks A.R., Medlock J.M., Johnson N. (2017). Emergence of *Babesia canis* in southern England. Parasit. Vectors.

[118] Cochez C., Lempereur L., Madder M., Claerebout E., Simons L., De Wilde N., Linden A., Saegerman C., Heyman P., Losson B. (2012). Foci report on indigenous *Dermacentor reticulatus* populations in Belgium and a preliminary study of associated babesiosis pathogens. Med. Vet. Entomol..

[119] Jongejan F., Ringenier M., Putting M., Berger L., Burgers S., Kortekaas R., Lenssen J., van Roessel M., Wijnveld M., Madder M. (2015). Novel foci of *Dermacentor reticulatus* tic.ks infected with *Babesia canis* and *Babesia caballi* in the Netherlands and in Belgium. Parasit. Vectors.

[120] Radzijevskaja J., Mardosaitė-Busaitienė D., Aleksandravičienė A., Paulauskas A. (2018). Investigation of *Babesia* spp. in sympatric populations of *Dermacentor reticulatus* and *Ixodes ricinus* ticks in Lithuania and Latvia. Ticks Tick Borne Dis..

[121] Levytska V.A., Mushinsky A.B., Zubrikova D., Blanarova L., Długosz E., Vichova B., Slivinska K.A., Gajewski Z., Gizinski S., Liu S. (2021). Detection of pathogens in ixodid ticks collected from animals and vegetation in five regions of Ukraine. Ticks Tick Borne Dis..

[122] Karbowiak G., Vichová B., Slivinska K., Werszko J., Didyk J., Peťko B., Stanko M., Akimov I. (2014). The infection of questing *Dermacentor reticulatus* ticks with *Babesia canis* and *Anaplasma phagocytophilum* in the Chernobyl exclusion zone. Vet. Parasitol..

[123] Daněk O., Hrazdilová K., Kozderková D., Jirků D., Modrý D. (2022). The distribution of *Dermacentor reticulatus* in the Czech Republic re-assessed: Citizen science approach to understanding the current distribution of the *Babesia canis* vector. Parasit. Vectors.

[124] Rar V.A., Maksimova T.G., Zakharenko L.P., Bolykhina S.A., Dobrotvorsky A.K., Morozova O.V. (2005). *Babesia* DNA detection in canine blood and *Dermacentor reticulatus* ticks in southwestern Siberia, Russia. Vector Borne Zoonotic Dis..

[125] Rar V.A., Fomenko N.V., Dobrotvorsky A.K., Livanova N.N., Rudakova S.A., Fedorov E.G., Astanin V.B., Morozova O.V. (2005). Tickborne Pathogen Detection, Western Siberia, Russia. Emerg. Infect. Dis..

[126] Tomanović S., Chochlakis D., Radulović Z., Milutinović M., Cakić S., Mihaljica D., Tselentis Y., Psaroulaki A. (2013). Analysis of pathogen co-occurrence in host-seeking adult hard ticks from Serbia. Exp. Appl. Acarol..

[127] Mihaljica D., Radulović Ž., Tomanović S., Ćakić S., Penezić A., Milutinović M. (2012). Molecular detection of *Babesia* spp. in ticks in northern Serbia. Arch. Biol. Sci..

[128] Hornok S., Kartali K., Takács N., Hofmann-Lehmann R. (2016). Uneven seasonal distribution of *Babesia canis* and its two 18S rDNA genotypes in questing *Dermacentor reticulatus* ticks in urban habitats. Ticks Tick Borne Dis..

[129] Villa L., Zanzani S.A., Mortarino M., Gazzonis A.L., Olivieri E., Manfredi M.T. (2022). Molecular Prevalence of Selected Tick-Borne Pathogens in *Dermacentor reticulatus* Collected in a Natural Park in Italy. Pathogens.

[130] Zygner W., Jaros S., Wędrychowicz H. (2008). Prevalence of *Babesia canis*, *Borrelia afzelii*, and *Anaplasma phagocytophilum* infection in hard ticks removed from dogs in Warsaw (central Poland). Vet. Parasitol..

[131] Schreiber C., Krücken J., Beck S., Maaz D., Pachnicke S., Krieger K., Gross M., Kohn B., von Samson-Himmelstjerna G. (2014). Pathogens in ticks collected from dogs in Berlin/Brandenburg, Germany. Parasit. Vectors.

[132] Abdullah S., Helps C., Tasker S., Newbury H., Wall R. (2018). Prevalence and distribution of *Borrelia* and *Babesia* species in ticks feeding on dogs in the U.K.. Med. Vet. Entomol..

[133] Nijhof A.M., Bodaan C., Postigo M., Nieuwenhuijs H., Opsteegh M., Franssen L., Jebbink F., Jongejan F. (2007). Ticks and associated pathogens collected from domestic animals in the Netherlands. Vector Borne Zoonotic Dis..

[134] Namina A., Capligina V., Seleznova M., Krumins R., Aleinikova D., Kivrane A., Akopjana S., Lazovska M., Berzina I., Ranka R. (2019). Tick-borne pathogens in ticks collected from dogs, Latvia, 2011–2016. BMC Vet. Res..

[135] Hamel D., Silaghi C., Zapadynska S., Kudrin A., Pfister K. (2013). Vector-borne pathogens in ticks and EDTA-blood samples collected from client-owned dogs, Kiev, Ukraine. Ticks Tick Borne Dis..

[136] Földvári G., Márialigeti M., Solymosi N., Lukács Z., Majoros G., Kósa J.P., Farkas R. (2007). Hard Ticks Infesting Dogs in Hungary and their Infection with *Babesia* and *Borrelia* Species. Parasitol. Res..

[137] Geurden T., Becskei C., Six R.H., Maeder S., Latrofa M.S., Otranto D., Farkas R. (2018). Detection of tick-borne pathogens in ticks from dogs and cats in different European countries. Ticks Tick Borne Dis..

[138] René-Martellet M., Moro C.V., Chêne J., Bourdoiseau G., Chabanne L., Mavingui P. (2015). Update on epidemiology of canine babesiosis in Southern France. BMC Vet. Res..

[139] Porchet M.J., Sager H., Muggli L., Oppliger A., Müller N., Frey C., Gottstein B. (2007). Etude épidémiologique descriptive de la Babésiose canine dans la Région Lémanique. Schweiz. Arch. Tierheilkd..

[140] Sukara R., Chochlakis D., Ćirović D., Penezić A., Mihaljica D., Ćakić S., Valčić M., Tselentis Y., Psaroulaki A., Tomanović S. (2018). Golden jackals (*Canis aureus*) as hosts for ticks and tick-borne pathogens in Serbia. Ticks Tick Borne Dis..

[141] Potkonjak A., Gutiérrez R., Savić S., Vračar V., Nachum-Biala Y., Jurišić A., Kleinerman G., Rojas A., Petrović A., Baneth G. (2016). Molecular detection of emerging tick-borne pathogens in Vojvodina, Serbia. Ticks Tick Borne Dis..

[142] Domatskiy V.N. (2022). Babesiosis of dogs distribution in the Russian Federation (review). Bull. KrasGAU.

[143] Zygner W., Górski P., Wędrychowicz H. (2009). Detection of the DNA of *Borrelia afzelii*, *Anaplasma phagocytophilum* and *Babesia canis* in blood samples from dogs in Warsaw. Vet. Rec..

[144] Welc-Falęciak R., Rodo A., Siński E., Bajer A. (2009). *Babesia canis* and other tick-borne infections in dogs in Central Poland. Vet Parasitol..

[145] Bajer A., Kowalec M., Levytska V.A., Mierzejewska E.J., Alsarraf M., Poliukhovych V., Rodo A., Wężyk D., Dwużnik-Szarek D. (2022). Tick-Borne Pathogens, *Babesia* spp. and *Borrelia burgdorferi* s.l., in Sled and Companion Dogs from Central and North-Eastern Europe. Pathogens.

[146] Adaszek Ł., Martinez A.C., Winiarczyk S. (2011). The factors affecting the distribution of babesiosis in dogs in Poland. Vet. Parasitol..

[147] Mierzejewska E.J., Dwużnik D., Koczwarska J., Stańczak Ł., Opalińska P., Krokowska-Paluszak M., Wierzbicka A., Górecki G., Bajer A. (2021). The red fox (*Vulpes vulpes*), a possible reservoir of *Babesia vulpes*, *B. canis* and *Hepatozoon canis* and its association with the tick *Dermacentor reticulatus* occurrence. Ticks Tick Borne Dis..

[148] Konvalinová J., Rudolf I., Šikutová S., Hubálek Z., Svobodová V., Svoboda M. (2012). Contribution to canine babesiosis in the Czech Republic. Acta Vet. Brno.

[149] Mitkova B., Hrazdilova K., Novotna M., Jurankova J., Hofmannova L., Forejtek P., Modry D. (2017). Autochthonous *Babesia canis*, *Hepatozoon canis* and imported *Babesia gibsoni* infection in dogs in the Czech Republic. Vet. Med..

[150] Kubelová M., Sedlák K., Panev A., Široký P. (2013). Conflicting results of serological, PCR and microscopic methods clarify the various risk levels of canine babesiosis in Slovakia: A complex approach to *Babesia canis* diagnostics. Vet. Parasitol..

[151] Víchová B., Miterpáková M., Iglódyová A. (2014). Molecular detection of co-infections with *Anaplasma phagocytophilum* and/or *Babesia canis canis* in *Dirofilaria*-positive dogs from Slovakia. Vet. Parasitol..

[152] Seleznova M., Kivrane A., Namina A., Krumins R., Aleinikova D., Lazovska M., Akopjana S., Capligina V., Ranka R. (2020). Babesiosis in Latvian domestic dogs, 2016–2019. Ticks Tick Borne Dis..

[153] Paulauskas A., Radzijevskaja J., Karvelienė B., Grigonis A., Aleksandravičienė A., Zamokas G., Babickaitė L., Sabūnas V., Petkevičius S. (2014). Detection and molecular characterization of canine babesiosis causative agent *Babesia canis* in the naturally infected dog in Lithuania. Vet. Parasitol..

[154] Ćoralić A., Gabrielli S., Zahirović A., Stojanović N.M., Milardi G.L., Jažić A., Zuko A., Čamo D., Otašević S. (2018). First molecular detection of *Babesia canis* in dogs from Bosnia and Herzegovina. Ticks Tick Borne Dis..

[155] Hodžić A., Alić A., Fuehrer H.P., Harl J., Wille-Piazzai W., Duscher G.G. (2015). A molecular survey of vector-borne pathogens in red foxes (*Vulpes vulpes*) from Bosnia and Herzegovina. Parasit. Vectors.

[156] Beck A., Huber D., Polkinghorne A., Kurilj A.G., Benko V., Mrljak V., Reljić S., Kusak J., Reil I., Beck R. (2017). The prevalence and impact of *Babesia canis* and *Theileria* sp. in free-ranging grey wolf (*Canis lupus*) populations in Croatia. Parasit. Vectors.

[157] Beck R., Vojta L., Mrljak V., Marinculić A., Beck A., Zivicnjak T., Cacciò S.M. (2009). Diversity of *Babesia* and *Theileria* species in symptomatic and asymptomatic dogs in Croatia. Int. J. Parasitol..

[158] Mrljak V., Kuleš J., Mihaljević Ž., Torti M., Gotić J., Crnogaj M., Živičnjak T., Mayer I., Šmit I., Bhide M. (2017). Prevalence and Geographic Distribution of Vector-Borne Pathogens in Apparently Healthy Dogs in Croatia. Vector Borne Zoonotic Dis..

[159] Kovačević Filipović M.M., Beletić A.D., Ilić Božović A.V., Milanović Z., Tyrrell P., Buch J., Breitschwerdt E.B., Birkenheuer A.J., Chandrashekar R. (2018). Molecular and Serological Prevalence of *Anaplasma phagocytophilum*, *A. platys*, *Ehrlichia canis*, *E. chaffeenses*, *E. ewingii*, *Borrelia burgdorferi*, *Babesia canis*, *B. gibsoni* and *B. vogeli* among Clinically Healthy Outdoor Dogs in Serbia. Vet. Parasitol. Reg. Stud. Rep..

[160] Duh D., Tozon N., Petrovec M., Strašek K., Avšič-Županc T. (2004). Canine babesiosis in Slovenia: Molecular evidence of *Babesia canis canis* and *Babesia canis vogeli*. Vet. Res..

[161] Hamel D., Silaghi C., Knaus M., Visser M., Kusi I., Rapti D., Rehbein S., Pfister K. (2009). Detection of *Babesia canis* subspecies and other arthropod-borne diseases in dogs from Tirana, Albania. Wien. Klin. Wochenschr..

[162] Pantchev N., Schnyder M., Vrhovec M.G., Schaper R., Tsachev I. (2015). Current Surveys of the Seroprevalence of *Borrelia burgdorferi*, *Ehrlichia canis*, *Anaplasma phagocytophilum*, *Leishmania infantum*, *Babesia canis*, *Angiostrongylus vasorum* and *Dirofilaria immitis* in Dogs in Bulgaria. Parasitol. Res..

[163] D’Amico G., Ionică A.M., Györke A., Dumitrache M.O. (2022). Epidemiological Survey of the Main Tick-Borne Pathogens Infecting Dogs from the Republic of Moldova. Pathogens.

[164] Cimpan A.A., Nachum-Biala Y., Ben-Shitrit B., Miron L., Baneth G. (2020). Epidemiological Study of Canine Babesiosis and Hepatozoonosis in the South of Romania. Acta Parasitol..

[165] Andersson M.O., Tolf C., Tamba P., Stefanache M., Waldenström J., Dobler G., Chițimia-Dobler L. (2017). Canine tick-borne diseases in pet dogs from Romania. Parasit. Vectors.

[166] Hamel D., Silaghi C., Lescai D., Pfister K. (2012). Epidemiological aspects on vector-borne infections in stray and pet dogs from Romania and Hungary with focus on *Babesia* spp.. Parasitol. Res..

[167] Mitková B., Hrazdilová K., D’Amico G., Duscher G.G., Suchentrunk F., Forejtek P., Gherman C.M., Matei I.A., Ionică A.M., Daskalaki A.A. (2017). Eurasian golden jackal as host of canine vector-borne protists. Parasit. Vectors.

[168] Hornok S., Edelhofer R., Farkas R. (2006). Seroprevalence of canine babesiosis in Hungary suggesting breed predisposition. Parasitol. Res..

[169] Menn B., Lorentz S., Naucke T.J. (2010). Imported and travelling dogs as carriers of canine vector-borne pathogens in Germany. Parasit. Vectors.

[170] Seibert S., Rohrberg A., Stockinger A., Schaalo S., März I. (2022). Occurrence of canine babesiosis in dogs in the Rhine-Main area of Hesse, Germany—A case study of 81 dogs. Tierarztl. Prax. Ausg. K Kleintiere Heimtiere.

[171] Pennisi M.G., Caprì A., Solano-Gallego L., Lombardo G., Torina A., Masucci M. (2012). Prevalence of antibodies against *Rickettsia conorii*, *Babesia canis*, *Ehrlichia canis*, and *Anaplasma phagocytophilum* antigens in dogs from the Stretto di Messina area (Italy). Ticks Tick Borne Dis..

[172] Ebani V.V., Nardoni S., Fognani G., Mugnaini L., Bertelloni F., Rocchigiani G., Papini R.A., Stefani F., Mancianti F. (2015). Molecular detection of vector-borne bacteria and protozoa in healthy hunting dogs from Central Italy. Asian Pac. J. Trop. Biomed..

[173] Veneziano V., Piantedosi D., Ferrari N., Neola B., Santoro M., Pacifico L., Sgroi G., D’Alessio N., Panico T., Leutenegger C.M. (2018). Distribution and risk factors associated with *Babesia* spp. infection in hunting dogs from Southern Italy. Ticks Tick Borne Dis..

[174] Solano-Gallego L., Trotta M., Carli E., Carcy B., Caldin M., Furlanello T. (2008). *Babesia canis canis* and *Babesia canis vogeli* clinicopathological findings and DNA detection by means of PCR-RFLP in blood from Italian dogs suspected of tick-borne disease. Vet. Parasitol..

[175] Aktas M., Özübek S., Altay K., Ipek N.D., Balkaya İ., Utuk A.E., Kırbas A., Şimsek S., Dumanlı N. (2015). Molecular detection of tick-borne rickettsial and protozoan pathogens in domestic dogs from Turkey. Parasit. Vectors.

[176] Khanmohammadi M., Zolfaghari-Emameh R., Arshadi M., Razmjou E., Karimi P. (2021). Molecular Identification and Genotyping of *Babesia canis* in Dogs from Meshkin Shahr County, Northwestern Iran. J. Arthropod Borne Dis..

[177] Wang J., Liu J., Yang J., Liu Z., Wang X., Li Y., Luo J., Guan G., Yin H. (2019). Molecular detection and genetic diversity of *Babesia canis canis* in pet dogs in Henan Province, China. Parasitol. Int..

[178] Ghasemzade M., Esmaeilnejad B., Asri-Rezaei S., Hadian M. (2022). Molecular identification of Babesia canis canis genotype A in a dog from Iran. Vet. Med. Sci..

[179] Aktas M., Ozubek S. (2017). A survey of canine haemoprotozoan parasites from Turkey, including molecular evidence of an unnamed *Babesia*. Comp. Immunol. Microbiol. Infect. Dis..

[180] Tiškina V., Capligina V., Must K., Berzina I., Ranka R., Jokelainen P. (2016). Fatal *Babesia canis canis* infection in a splenectomized Estonian dog. Acta Vet. Scand..

[181] Kamani J., Sannusi A., Dogo A.G., Tanko J.T., Egwu K.O., Tafarki A.E., Ogo I.N., Kemza S., Onovoh E., Shamaki D. (2010). *Babesia canis* and *Babesia rossi* co-infection in an untraveled Nigerian dog. Vet. Parasitol..

[182] Tayyub M., Ashraf K., Lateef M., Anjum A.A., Ali M.A., Ahmad N., Nawaz M., Nazir M.M. (2019). Genetic Diversity of Canine *Babesia* Species Prevalent in Pet Dogs of Punjab, Pakistan. Animals.

[183] Zanet S., Bassano M., Trisciuoglio A., Taricco I., Ferroglio E. (2017). Horses infected by Piroplasms different from *Babesia caballi* and *Theileria equi*: Species identification and risk factors analysis in Italy. Vet. Parasitol..

[184] Vilhena H., Martinez-Díaz V.L., Cardoso L., Vieira L., Altet L., Francino O., Pastor J., Silvestre-Ferreira A.C. (2013). Feline vector-borne pathogens in the north and centre of Portugal. Parasit. Vectors.

[185] Hornok S., Estók P., Kováts D., Flaisz B., Takács N., Szőke K., Krawczyk A., Kontschán J., Gyuranecz M., Fedák A. (2015). Screening of bat faeces for arthropod-borne apicomplexan protozoa: *Babesia canis* and *Besnoitia besnoiti*-like sequences from Chiroptera. Parasit. Vectors.

[186] Corduneanu A., Ursache T.D., Taulescu M., Sevastre B., Modrý D., Mihalca A.D. (2020). Detection of DNA of *Babesia canis* in tissues of laboratory rodents following oral inoculation with infected ticks. Parasit. Vectors.

[187] Kirman R., Guven E. (2023). Molecular detection of *Babesia* and *Theileria* species/genotypes in sheep and ixodid ticks in Erzurum, Northeastern Turkey: First report of *Babesia canis* in sheep. Res. Vet. Sci..

[188] Hornok S., Edelhofer R., Földvári G., Joachim A., Farkas R. (2007). Serological evidence for *Babesia canis* infection of horses and an endemic focus of *B. caballi* in Hungary. Acta Vet. Hung..

[189] Dantas-Torres F., Latrofa M.S., Annoscia G., Giannelli A., Parisi A., Otranto D. (2013). Morphological and genetic diversity of *Rhipicephalus sanguineus* sensu lato from the New and Old Worlds. Parasit. Vectors.

[190] Dantas-Torres F., Otranto D. (2015). Further thoughts on the taxonomy and vector role of *Rhipicephalus sanguineus* group ticks. Vet. Parasitol..

[191] Mihalca A.D., Kalmár Z., Dumitrache M.O. (2015). *Rhipicephalus rossicus*, a neglected tick at the margin of Europe: A review of its distribution, ecology and medical importance. Med. Vet. Entomol..

[192] Bakkes D.K., Chitimia-Dobler L., Matloa D., Oosthuysen M., Mumcuoglu K.Y., Mans B.J., Matthee C.A. (2020). Integrative taxonomy and species delimitation of *Rhipicephalus turanicus* (Acari: Ixodida: Ixodidae). Int. J. Parasitol..

[193] European Centre for Disease Prevention and Control. https://www.ecdc.europa.eu/en/disease-vectors/surveillance-and-disease-data/tick-maps.

[194] Rubel F., Dautel H., Nijhof A.M., Kahl O. (2022). Ticks in the metropolitan area of Berlin, Germany. Ticks Tick Borne Dis..

[195] Hansford K.M., Phipps L.P., Cull B., Pietzsch M.E., Medlock J.M. (2017). *Rhipicephalus sanguineus* importation into the UK: Surveillance, risk, public health awareness and One Health response. Vet. Rec..

[196] Richter S.H., Eydal M., Skírnisson K., Ólafsson E. (2013). Tick species (Ixodida) identified in Iceland. Icel. Agric. Sci..

[197] Nowak-Chmura M. (2014). A biological/medical review of alien tick species (Acari: Ixodida) accidentally transferred to Poland. Ann. Parasitol..

[198] Nava S., Beati L., Venzal J.M., Labruna M.B., Szabó M.P.J., Petney T., Saracho-Bottero M.N., Tarragona E.L., Dantas-Torres F., Silva M.M.S. (2018). *Rhipicephalus sanguineus* (Latreille, 1806): Neotype designation, morphological re-description of all parasitic stages and molecular characterization. Ticks Tick Borne Dis..

[199] Jones E.O., Gruntmeir J.M., Hamer S.A., Little S.E. (2017). Temperate and tropical lineages of brown dog ticks in North America. Vet. Parasitol. Reg. Stud. Rep..

[200] Pascoe E.L., Nava S., Labruna M.B., Paddock C.D., Levin M.L., Marcantonio M., Foley J.E. (2022). Predicting the northward expansion of tropical lineage *Rhipicephalus sanguineus* sensu lato ticks in the United States and its implications for medical and veterinary health. PLoS ONE.

[201] Grant A.N., Lineberry M.W., Sundstrom K.D., Allen K.E., Little S.E. (2023). Geographic Distribution and Seasonality of Brown Dog Tick Lineages in the United States. J. Med. Entomol..

[202] Chandra S., Ma G.C., Burleigh A., Brown G., Norris J.M., Ward M.P., Emery D., Šlapeta J. (2020). The brown dog tick *Rhipicephalus sanguineus* sensu Roberts, 1965 across Australia: Morphological and molecular identification of *R. sanguineus* s.l. tropical lineage. Ticks Tick Borne Dis..

[203] Roberts F.H.S. (1965). The taxonomic status of the species of the genera *Rhipicephalus* Koch and *Boophilus* Curtice (Acarina: Ixodidae) occurring in Australia. Aust. J. Zool..

[204] Šlapeta J., Chandra S., Halliday B. (2021). The “tropical lineage” of the brown dog tick *Rhipicephalus sanguineus* sensu lato identified as *Rhipicephalus linnaei* (Audouin, 1826). Int. J. Parasitol..

[205] Šlapeta J., Halliday B., Chandra S., Alanazi A.D., Abdel-Shafy S. (2022). *Rhipicephalus linnaei* (Audouin, 1826) recognised as the “tropical lineage” of the brown dog tick *Rhipicephalus sanguineus* sensu lato: Neotype designation, redescription, and establishment of morphological and molecular reference. Ticks Tick Borne Dis..

[206] Greay T.L., Zahedi A., Krige A.S., Owens J.M., Rees R.L., Ryan U.M., Oskam C.L., Irwin P.J. (2018). Endemic, exotic and novel apicomplexan parasites detected during a national study of ticks from companion animals in Australia. Parasit. Vectors.

[207] Brown G.K., Canfield P.J., Dunstan R.H., Roberts T.K., Martin A.R., Brown C.S., Irving R. (2006). Detection of *Anaplasma platys* and *Babesia canis vogeli* and their impact on platelet numbers in free-roaming dogs associated with remote Aboriginal communities in Australia. Aust. Vet. J..

[208] Hii S.F., Kopp S.R., Thompson M.F., O’Leary C.A., Rees R.L., Traub R.J. (2012). Canine vector-borne disease pathogens in dogs from south-east Queensland and north-east Northern Territory. Aust. Vet. J..

[209] Hii S.F., Traub R.J., Thompson M.F., Henning J., O’Leary C.A., Burleigh A., McMahon S., Rees R.L., Kopp S.R. (2015). Canine tick-borne pathogens and associated risk factors in dogs presenting with and without clinical signs consistent with tick-borne diseases in northern Australia. Aust. Vet. J..

[210] Šlapeta J., Halliday B., Dunlop J.A., Nachum-Biala Y., Salant H., Ghodrati S., Modrý D., Harrus S. (2023). The “southeastern Europe” lineage of the brown dog tick *Rhipicephalus sanguineus* (sensu lato) identified as *Rhipicephalus rutilus* Koch, 1844: Comparison with holotype and generation of mitogenome reference from Israel. Curr. Res. Parasitol. Vector Borne Dis..

[211] Sprong H., Fonville M., Docters van Leeuwen A., Devillers E., Ibañez-Justicia A., Stroo A., Hansford K., Cull B., Medlock J., Heyman P. (2019). Detection of pathogens in *Dermacentor reticulatus* in northwestern Europe: Evaluation of a high-throughput array. Heliyon.

[212] Zheng W.Q., Xuan X.N., Fu R.L., Tao H.Y., Liu Y.Q., Liu X.Q., Li D.M., Ma H.M., Chen H.Y. (2018). Tick-Borne Pathogens in Ixodid Ticks from Poyang Lake Region, Southeastern China. Korean J. Parasitol..

[213] Wei F.R., Lan Q.X., Zhu D., Ye J.H., Liu Q., Zhang Y. (2012). Investigation on *Babesia* in ticks infested on police dogs in selected areas of China. Zhongguo Ji Sheng Chong Xue Yu Ji Sheng Chong Bing Za Zhi.

[214] Laatamna A., Strube C., Bakkes D.K., Schaper S., Aziza F.Z., Ben Chelef H., Amrane N.E.H., Bedraoui R., Dobler G., Chitimia-Dobler L. (2022). Molecular detection of tick-borne pathogens in *Rhipicephalus sanguineus* sensu stricto collected from dogs in the steppe and high plateau regions of Algeria. Acta Trop..

[215] Melo A.L.T., Witter R., Martins T.F., Pacheco T.A., Alves A.S., Chitarra C.S., Dutra V., Nakazato L., Pacheco R.C., Labruna M.B. (2016). A survey of tick-borne pathogens in dogs and their ticks in the Pantanal biome, Brazil. Med. Vet. Entomol..

[216] Araujo A.C., Silveira J.A.G., Azevedo S.S., Nieri-Bastos F.A., Ribeiro M.F.B., La-bruna M.B., Horta M.C. (2015). *Babesia canis vogeli* infection in dogs and ticks in the semiarid region of Pernambuco, Brazil. Pesq. Vet. Bras..

[217] Panti-May J.A., Rodríguez-Vivas R.I. (2020). Canine babesiosis: A literature review of prevalence, distribution, and diagnosis in Latin America and the Caribbean. Vet. Parasitol. Reg. Stud. Rep..

[218] Lira-Amaya J.J., Rojas-Martínez C., Álvarez-Martínez A., Pelaez-Flores A., Martínez-Ibañez F., Perez de la Rosa D., Figueroa-Millan J.V. (2017). First Molecular Detection of *Babesia canis vogeli* in Dogs and *Rhipicephalus sanguineus* from Mexico. Arch. Palliat. Care.

[219] Reeves W.K., Wolf S., Rabago R., Gutierrez T., Nunn P., Johnson J., Vice D. (2012). Invertebrate Vectors, Parasites, and Rickettsial Agents in Guam. Micronesica.

[220] Harrus S., Perlman-Avrahami A., Mumcuoglu K.Y., Morick D., Eyal O., Baneth G. (2011). Molecular detection of *Ehrlichia canis*, *Anaplasma bovis*, *Anaplasma platys*, *Candidatus* Midichloria mitochondrii and *Babesia canis vogeli* in ticks from Israel. Clin. Microbiol. Infect..

[221] Mumcuoglu K.Y., Arslan-Akveran G., Aydogdu S., Karasartova D., Koşar A., Savci U., Keskin A., Taylan-Ozkan A. (2022). Pathogens in ticks collected in Israel: II. Bacteria and protozoa found in *Rhipicephalus sanguineus* sensu lato and *Rhipicephalus turanicus*. Ticks Tick Borne Dis..

[222] Maia C., Ferreira A., Nunes M., Vieira M.L., Campino L., Cardoso L. (2014). Molecular detection of bacterial and parasitic pathogens in hard ticks from Portugal. Ticks Tick Borne Dis..

[223] Millán J., Proboste T., Fernández de Mera I.G., Chirife A.D., de la Fuente J., Altet L. (2016). Molecular detection of vector-borne pathogens in wild and domestic carnivores and their ticks at the human–wildlife interface. Ticks Tick Borne Dis..

[224] Zanet S., Battisti E., Pepe P., Ciuca L., Colombo L., Trisciuoglio A., Ferroglio E., Cringoli G., Rinaldi L., Maurelli M.P. (2020). Tick-borne pathogens in Ixodidae ticks collected from privately-owned dogs in Italy: A country-wide molecular survey. BMC Vet. Res..

[225] Azmi K., Al-Jawabreh A., Nasereddin A., Abdelkader A., Zaid T., Ereqat S., Sawalha S.S., Baneth G., Abdeen Z. (2017). Detection and molecular identification of *Hepatozoon canis* and *Babesia vogeli* from domestic dogs in Palestine. Parasitology.

[226] Manoj R.R.S., Iatta R., Latrofa M.S., Capozzi L., Raman M., Colella V., Otranto D. (2020). Canine vector-borne pathogens from dogs and ticks from Tamil Nadu, India. Acta Trop..

[227] Prakash B.K., Low V.L., Vinnie-Siow W.Y., Tan T.K., Lim Y.A., Morvarid A.R., AbuBakar S., Sofian-Azirun M. (2018). Detection of *Babesia* spp. in Dogs and Their Ticks From Peninsular Malaysia: Emphasis on *Babesia gibsoni* and *Babesia vogeli* Infections in *Rhipicephalus sanguineus* sensu lato (Acari: Ixodidae). J. Med. Entomol..

[228] Nguyen V.L., Colella V., Greco G., Fang F., Nurcahyo W., Hadi U.K., Venturina V., Tong K.B.Y., Tsai Y.L., Taweethavonsawat P. (2020). Molecular detection of pathogens in ticks and fleas collected from companion dogs and cats in East and Southeast Asia. Parasit. Vectors.

[229] Jongejan F., Su B.L., Yang H.J., Berger L., Bevers J., Liu P.C., Fang J.C., Cheng Y.W., Kraakman C., Plaxton N. (2018). Molecular evidence for the transovarial passage of *Babesia gibsoni* in *Haemaphysalis hystricis* (Acari: Ixodidae) ticks from Taiwan: A novel vector for canine babesiosis. Parasit. Vectors.

[230] Galay R.L., Manalo A.A.L., Dolores S.L.D., Aguilar I.P.M., Sandalo K.A.C., Cruz K.B., Divina B.P., Andoh M., Masatani T., Tanaka T. (2018). Molecular detection of tick-borne pathogens in canine population and *Rhipicephalus sanguineus* (sensu lato) ticks from southern Metro Manila and Laguna, Philippines. Parasit. Vectors.

[231] Nguyen V.L., Colella V., Iatta R., Bui K.L., Dantas-Torres F., Otranto D. (2019). Ticks and associated pathogens from dogs in northern Vietnam. Parasitol. Res..

[232] Huynh L.N., Diarra A.Z., Pham Q.L., Le-Viet N., Berenger J.M., Ho V.H., Nguyen X.Q., Parola P. (2021). Morphological, molecular and MALDI-TOF MS identification of ticks and tick-associated pathogens in Vietnam. PLoS Negl. Trop. Dis..

[233] Xu D., Zhang J., Shi Z., Song C., Zheng X., Zhang Y., Hao Y., Dong H., Wei L., El-Mahallawy H.S. (2015). Molecular detection of vector-borne agents in dogs from ten provinces of China. Parasit. Vectors.

[234] Hegab A.A., Omar H.M., Abuowarda M., Ghattas S.G., Mahmoud N.E., Fahmy M.M. (2022). Screening and phylogenetic characterization of tick-borne pathogens in a population of dogs and associated ticks in Egypt. Parasit. Vectors.

[235] M’ghirbi Y., Bouattour A. (2008). Detection and molecular characterization of *Babesia canis vogeli* from naturally infected dogs and *Rhipicephalus sanguineus* ticks in Tunisia. Vet. Parasitol..

[236] Barradas P.F., Mesquita J.R., Ferreira P., Amorim I., Gärtner F. (2020). Detection of tick-borne pathogens in *Rhipicephalus sanguineus* sensu lato and dogs from different districts of Portugal. Ticks Tick Borne Dis..

[237] Habibi G., Imani A., Afshari A., Bozorgi S. (2020). Detection and Molecular Characterization of *Babesia canis vogeli* and *Theileria annulata* in Free-Ranging Dogs and Ticks from Shahriar County, Tehran Province, Iran. Iran. J. Parasitol..

[238] Ribeiro C.M., Matos A.C., Azzolini T., Bones E.R., Wasnieski E.A., Richini-Perera V.B., Lucheis S.B., Vidotto O. (2017). Molecular epidemiology of *Anaplasma platys*, *Ehrlichia canis* and *Babesia vogeli* in stray dogs in Paraná, Brazil. Pesq. Vet. Bras..

[239] Paulino P.G., Pires M.S., da Silva C.B., Peckle M., da Costa R.L., Vitari G.L.V., de Abreu A.P.M., Massard C.L., Santos H.A. (2018). Molecular epidemiology of *Babesia vogeli* in dogs from the southeastern region of Rio de Janeiro, Brazil. Vet. Parasitol. Reg. Stud. Rep..

[240] Costa-Júnior L.M., Zahler-Rinder M., Ribeiro M.F., Rembeck K., Rabelo E.M., Pfister K., Passos L.M. (2012). Use of a Real Time PCR for detecting subspecies of *Babesia canis*. Vet. Parasitol..

[241] Rotondano T.E., Almeida H.K., Krawczak Fda S., Santana V.L., Vidal I.F., Labruna M.B., de Azevedo S.S., Ade lmeida A.M., de Melo M.A. (2015). Survey of *Ehrlichia canis*, *Babesia* spp. and *Hepatozoon* spp. in dogs from a semiarid region of Brazil. Rev. Bras. Parasitol. Vet..

[242] Thomas R.S., Santodomingo A.M., Castro L.R. (2020). Molecular detection of *Babesia canis vogeli* and *Hepatozoon canis* in dogs in the department of Magdalena (Colombia). Rev. Med. Vet. Zoot..

[243] García-Quesada A., Jiménez-Rocha A., Romero-Zuñiga J.J., Dolz G. (2021). Seroprevalence and prevalence of *Babesia vogeli* in clinically healthy dogs and their ticks in Costa Rica. Parasit. Vectors.

[244] Obeta S.S., Ibrahim B., Lawal I.A., Natala J.A., Ogo N.I., Balogun E.O. (2020). Prevalence of canine babesiosis and their risk factors among asymptomatic dogs in the federal capital territory, Abuja, Nigeria. Parasite Epidemiol. Control.

[245] Su B.L., Liu P.C., Fang J.C., Jongejan F. (2023). Correlation between *Babesia* Species Affecting Dogs in Taiwan and the Local Distribution of the Vector Ticks. Vet. Sci..

[246] Shapiro A.J., Brown G., Norris J.M., Bosward K.L., Marriot D.J., Balakrishnan N., Breitschwerdt E.B., Malik R. (2017). Vector-borne and zoonotic diseases of dogs in North-west New South Wales and the Northern Territory, Australia. BMC Vet. Res..

[247] Barker E.N., Langton D.A., Helps C.R., Brown G., Malik R., Shaw S.E., Tasker S. (2012). Haemoparasites of free-roaming dogs associated with several remote Aboriginal communities in Australia. BMC Vet. Res..

[248] Jain J., Lakshmanan B., Nagaraj H.V., Praveena J.E., Syamala K., Aravindakshan T. (2018). Detection of *Babesia canis vogeli*, *Babesia gibsoni* and *Ehrlichia canis* by multiplex PCR in naturally infected dogs in South India. Vet. Arh..

[249] Augustine S., Sabu L., Lakshmanan B. (2017). Molecular identification of *Babesia* spp. in naturally infected dogs of Kerala, South India. J. Parasit. Dis..

[250] Mittal M., Kundu K., Chakravarti S., Mohapatra J.K., Singh V.K., Raja Kumar B., Thakur V., Churamani C.P., Kumar A. (2019). Canine babesiosis among working dogs of organised kennels in India: A comprehensive haematological, biochemical, clinicopathological and molecular epidemiological multiregional study. Prev. Vet. Med..

[251] Díaz-Regañón D., Agulla B., Piya B., Fernández-Ruiz N., Villaescusa A., García-Sancho M., Rodríguez-Franco F., Sainz Á. (2020). Stray dogs in Nepal have high prevalence of vector-borne pathogens: A molecular survey. Parasit. Vectors.

[252] Iatta R., Sazmand A., Nguyen V.L., Nemati F., Ayaz M.M., Bahiraei Z., Zafari S., Giannico A., Greco G., Dantas-Torres F. (2021). Vector-borne pathogens in dogs of different regions of Iran and Pakistan. Parasitol. Res..

[253] Li X.W., Zhang X.L., Huang H.L., Li W.J., Wang S.J., Huang S.J., Shao J.W. (2020). Prevalence and molecular characterization of *Babesia* in pet dogs in Shenzhen, China. Comp. Immunol. Microbiol. Infect. Dis..

[254] Niu Q., Yang J., Liu Z., Gao S., Pan Y., Guan G., Chu Y., Liu G., Luo J., Yin H. (2017). First Molecular Detection of Piroplasm Infection in Pet Dogs from Gansu, China. Front. Microbiol..

[255] Sang C., Yang Y., Dong Q., Xu B., Liu G., Hornok S., Liu Z., Wang Y., Hazihan W. (2021). Molecular survey of *Babesia* spp. in red foxes (*Vulpes Vulpes*), Asian badgers (*Meles leucurus*) and their ticks in China. Ticks Tick Borne Dis..

[256] Muguiro D.H., Nekouei O., Lee K.Y., Hill F., Barrs V.R. (2023). Prevalence of *Babesia* and *Ehrlichia* in owned dogs with suspected tick-borne infection in Hong Kong, and risk factors associated with *Babesia gibsoni*. Prev. Vet. Med..

[257] Chandra S., Smith K., Alanazi A.D., Alyousif M.S., Emery D., Šlapeta J. (2019). *Rhipicephalus sanguineus* sensu lato from dogs and dromedary camels in Riyadh, Saudi Arabia: Low prevalence of vector-borne pathogens in dogs detected using multiplexed tandem PCR panel. Folia Parasitol..

[258] Alanazi A.D., Alouffi A.S., Alyousif M.S., Alshahrani M.Y., Abdullah H.H.A.M., Abdel-Shafy S., Calvani N.E.D., Ansari-Lari M., Sazmand A., Otranto D. (2021). Molecular Survey of Vector-Borne Pathogens of Dogs and Cats in Two Regions of Saudi Arabia. Pathogens.

[259] Piratae S., Pimpjong K., Vaisusuk K., Chatan W. (2015). Molecular detection of *Ehrlichia canis*, *Hepatozoon canis* and *Babesia canis vogeli* in stray dogs in Mahasarakham province, Thailand. Ann. Parasitol..

[260] Juasook A., Siriporn B., Nopphakhun N., Phetpoang P., Khamyang S. (2021). Molecular detection of tick-borne pathogens in infected dogs associated with *Rhipicephalus sanguineus* tick infestation in Thailand. Vet. World.

[261] Rucksaken R., Maneeruttanarungroj C., Maswanna T., Sussadee M., Kanbutra P. (2019). Comparison of conventional polymerase chain reaction and routine blood smear for the detection of *Babesia canis*, *Hepatozoon canis*, *Ehrlichia canis*, and *Anaplasma platys* in Buriram Province, Thailand. Vet. World.

[262] Luong N.H., Kamyingkird K., Thammasonthijarern N., Phasuk J., Nimsuphan B., Pattanatanang K., Chimnoi W., Kengradomkij C., Klinkaew N., Inpankaew T. (2023). Companion Vector-Borne Pathogens and Associated Risk Factors in Apparently Healthy Pet Animals (Dogs and Cats) in Khukhot City Municipality, Pathum Thani Province, Thailand. Pathogens.

[263] Sontigun N., Boonhoh W., Fungwithaya P., Wongtawan T. (2022). Multiple blood pathogen infections in apparently healthy sheltered dogs in southern Thailand. Int. J. Vet. Sci. Med..

[264] Inokuma H., Yoshizaki Y., Matsumoto K., Okuda M., Onishi T., Nakagome K., Kosugi R., Hirakawa M. (2004). Molecular survey of *Babesia* infection in dogs in Okinawa, Japan. Vet. Parasitol..

[265] Adao D.E.V., Herrera C.M.T., Galarion L.H., Bolo N.R., Carlos R.S., Carlos E.T., Carlos S.S., Rivera W.L. (2017). Detection and molecular characterization of *Hepatozoon canis*, *Babesia vogeli*, *Ehrlichia canis*, and *Anaplasma platys* in dogs from Metro Manila, Philippines. Korean J. Vet. Res..

[266] Inpankaew T., Hii S.F., Chimnoi W., Traub R.J. (2016). Canine vector-borne pathogens in semi-domesticated dogs residing in northern Cambodia. Parasit. Vectors.

[267] Selim A., Megahed A., Ben Said M., Alanazi A.D., Sayed-Ahmed M.Z. (2022). Molecular survey and phylogenetic analysis of *Babesia vogeli* in dogs. Sci. Rep..

[268] Zaki A.A., Attia M.M., Ismael E., Mahdy O.A. (2021). Prevalence, genetic, and biochemical evaluation of immune response of police dogs infected with *Babesia vogeli*. Vet. World.

[269] Cardoso L., Oliveira A.C., Granada S., Nachum-Biala Y., Gilad M., Lopes A.P., Sousa S.R., Vilhena H., Baneth G. (2016). Molecular investigation of tick-borne pathogens in dogs from Luanda, Angola. Parasit. Vectors.

[270] Matjila P.T., Penzhorn B.L., Bekker C.P., Nijhof A.M., Jongejan F. (2004). Confirmation of occurrence of *Babesia canis vogeli* in domestic dogs in South Africa. Vet. Parasitol..

[271] Gabrielli S., Otašević S., Ignjatović A., Savić S., Fraulo M., Arsić-Arsenijević V., Momčilović S., Cancrini G. (2015). Canine Babesioses in Noninvestigated Areas of Serbia. Vector Borne Zoonotic Dis..

[272] Licari E., Takács N., Solymosi N., Farkas R. (2017). First detection of tick-borne pathogens of dogs from Malta. Ticks Tick Borne Dis..

[273] Criado-Fornelio A., Rey-Valeiron C., Buling A., Barba-Carretero J.C., Jefferies R., Irwin P. (2007). New advances in molecular epizootiology of canine hematic protozoa from Venezuela, Thailand and Spain. Vet. Parasitol..

[274] Dordio A.M., Beck R., Nunes T., Pereira da Fonseca I., Gomes J. (2021). Molecular survey of vector-borne diseases in two groups of domestic dogs from Lisbon, Portugal. Parasit. Vectors.

[275] Kartashov S.N., Kolesnikov A.G., Butenkov A.I., Kartashova E.V. (2015). Vector dogs infection, clinical and morphological aspects of babesiosis in dogs in the Rostov region. Vet. Patol..

[276] Birkenheuer A.J., Correa M.T., Levy M.G., Breitschwerdt E.B. (2005). Geographic distribution of babesiosis among dogs in the United States and association with dog bites: 150 cases (2000–2003). J. Am. Vet. Med. Assoc..

[277] Javeed N.N., Shultz L., Barnum S., Foley J.E., Hodzic E., Pascoe E.L., Martínez-López B., Quinn N., Bucklin D., Dear J.D. (2022). Prevalence and geographic distribution of *Babesia conradae* and detection of *Babesia vogeli* in free-ranging California coyotes (*Canis latrans*). Int. J. Parasitol. Parasites Wildl..

[278] Kidd L., Qurollo B., Lappin M., Richter K., Hart J.R., Hill S., Osmond C., Breitschwerdt E.B. (2017). Prevalence of Vector-Borne Pathogens in Southern California Dogs with Clinical and Laboratory Abnormalities Consistent With Immune-Mediated Disease. J. Vet. Intern. Med..

[279] Yu S., Modarelli J., Tomeček J.M., French J.T., Hilton C., Esteve-Gasent M.D. (2020). Prevalence of common tick-borne pathogens in white-tailed deer and coyotes in south Texas. Int. J. Parasitol. Parasites Wildl..

[280] Tovar R.E.M., Flores R.A.N., Hernández I.V.N., Pérez L.E.R. (2019). Detección molecular de Anaplasma platys, Babesia spp., Ehrlichia canis y Hepatozoon canis en caninos (*Canis lupus* familiaris) con sospecha de hemoparásitos en clínicas veterinarias de Santa Tecla y San Salvador, El Salvador. Rev. Agrocenc..

[281] Wei L., Kelly P., Ackerson K., Zhang J., El-Mahallawy H.S., Kaltenboeck B., Wang C. (2014). First report of *Babesia gibsoni* in Central America and survey for vector-borne infections in dogs from Nicaragua. Parasit. Vectors.

[282] Starkey L.A., Newton K., Brunker J., Crowdis K., Edourad E.J.P., Meneus P., Little S.E. (2016). Prevalence of vector-borne pathogens in dogs from Haiti. Vet. Parasitol..

[283] Wei L., Kelly P., Ackerson K., El-Mahallawy H.S., Kaltenboeck B., Wang C. (2014). Molecular detection of *Dirofilaria immitis*, *Hepatozoon canis*, *Babesia* spp., *Anaplasma platys*, and *Ehrlichia canis* in dogs on Costa Rica. Acta Parasitol..

[284] Kelly P.J., Xu C., Lucas H., Loftis A., Abete J., Zeoli F., Stevens A., Jaegersen K., Ackerson K., Gessner A. (2013). Ehrlichiosis, Babesiosis, Anaplasmosis and Hepatozoonosis in Dogs from St. Kitts, West Indies. PLoS ONE.

[285] Yabsley M.J., McKibben J., Macpherson C.N., Cattan P.F., Cherry N.A., Hegarty B.C., Breitschwerdt E.B., O’Connor T., Chandrashekar R., Paterson T. (2008). Prevalence of *Ehrlichia canis*, *Anaplasma platys*, *Babesia canis vogeli*, *Hepatozoon canis*, *Bartonella vinsonii berkhoffii*, and *Rickettsia* spp. in dogs from Grenada. Vet. Parasitol..

[286] Georges K., Ezeokoli C.D., Newaj-Fyzul A., Campbell M., Mootoo N., Mutani A., Sparagano O.A. (2008). The Application of PCR and Reverse Line Blot Hybridization to Detect Arthropod-borne Hemopathogens of Dogs and Cats in Trinidad. Ann. N. Y. Acad. Sci..

[287] Vargas-Hernández G., André M.R., Faria J.L., Munhoz T.D., Hernandez-Rodriguez M., Machado R.Z., Tinucci-Costa M. (2012). Molecular and serological detection of *Ehrlichia canis* and *Babesia vogeli* in dogs in Colombia. Vet. Parasitol..

[288] Galván C., Miranda J., Mattar S., Ballut J. (2018). *Babesia* spp. in dogs from Córdoba, Colombia. Kafkas Univ. Vet. Fak. Derg..

[289] Levy J.K., Crawford P.C., Lappin M.R., Dubovi E.J., Levy M.G., Alleman R., Tucker S.J., Clifford E.L. (2008). Infectious Diseases of Dogs and Cats on Isabela Island, Galapagos. J. Vet. Intern. Med..

[290] Furtado M.M., Taniwaki S.A., Metzger B., Dos Santos Paduan K., O’Dwyer H.L., de Almeida Jácomo A.T., Porfírio G.E.O., Silveira L., Sollmann R., Tôrres N.M. (2017). Is the free-ranging jaguar (*Panthera onca*) a reservoir for *Cytauxzoon felis* in Brazil?. Ticks Tick Borne Dis..

[291] da Silva V.C.L., de Lima E.R., Dias M.B.D.M.C., Fukahori F.L.P., de Azevedo Rêgo M.S., Júnior J.W.P., Kim P.D.C.P., Leitão R.S.C.S., Mota R.A., de Oliveira Carieli E.P. (2016). Parasitological and molecular detection of *Babesia canis vogeli* in dogs of Recife, Pernambuco and evaluation of risk factors associated. Semin. Cienc. Agrar..

[292] de Sousa K.C.M., Fernandes M.P., Herrera H.M., Freschi C.R., Machado R.Z., André M.R. (2018). Diversity of piroplasmids among wild and domestic mammals and ectoparasites in Pantanal wetland, Brazil. Ticks Tick Borne Dis..

[293] Costa-Júnior L.M., Ribeiro M.F., Rembeck K., Rabelo E.M., Zahler-Rinder M., Hirzmann J., Pfister K., Passos L.M. (2009). Canine babesiosis caused by *Babesia canis vogeli* in rural areas of the State of Minas Gerais, Brazil and factors associated with its seroprevalence. Res. Vet. Sci..

[294] Vieira F.T., Acosta I.C.L., Martins T.F., Filho J.M., Krawczak F.D.S., Barbieri A.R.M., Egert L., Fernandes D.R., Braga F.R., Labruna M.B. (2018). Tick-borne infections in dogs and horses in the state of Espírito Santo, Southeast Brazil. Vet. Parasitol..

[295] Inácio E.L., Pérez-Macchi S., Alabi A., Bittencourt P., Müller A. (2019). Prevalence and molecular characterization of piroplasmids in domestic dogs from Paraguay. Ticks Tick Borne Dis..

[296] Temoche L.C., Assad R., Seabra-Junior E.S., Lemos T.D., Almosny N. (2018). Frequency of *Babesia vogeli* in domestic dogs in the metropolitan area of Piura, Peru. Acta Vet. Brno.

[297] Mascarelli P.E., Tartara G.P., Pereyra N.B., Maggi R.G. (2016). Detection of *Mycoplasma haemocanis*, *Mycoplasma haematoparvum*, *Mycoplasma suis* and other vector-borne pathogens in dogs from Córdoba and Santa Fé, Argentina. Parasit. Vectors.

[298] Millán J., Travaini A., Cevidanes A., Sacristán I., Rodríguez A. (2018). Assessing the natural circulation of canine vector-borne pathogens in foxes, ticks and fleas in protected areas of Argentine Patagonia with negligible dog participation. Int. J. Parasitol. Parasites Wildl..

[299] Di Cataldo S., Ulloa-Contreras C., Cevidanes A., Hernández C., Millán J. (2020). *Babesia vogeli* in dogs in Chile. Transbound. Emerg. Dis..

[300] Simking P., Wongnakphet S., Stich R.W., Jittapalapong S. (2010). Detection of *Babesia vogeli* in stray cats of metropolitan Bangkok, Thailand. Vet. Parasitol..

[301] Palmer J.P., Gazêta G., André M., Coelho A., Corrêa L., Damasceno J., Israel C., Pereira R., Barbosa A. (2022). Piroplasm Infection in Domestic Cats in the Mountainous Region of Rio de Janeiro, Brazil. Pathogens.

[302] Kelly P., Marabini L., Dutlow K., Zhang J., Loftis A., Wang C. (2014). Molecular detection of tick-borne pathogens in captive wild felids, Zimbabwe. Parasit. Vectors.

[303] Krücken J., Czirják G.Á., Ramünke S., Serocki M., Heinrich S.K., Melzheimer J., Costa M.C., Hofer H., Aschenborn O.H.K., Barker N.A. (2021). Genetic diversity of vector-borne pathogens in spotted and brown hyenas from Namibia and Tanzania relates to ecological conditions rather than host taxonomy. Parasit. Vectors.

[304] Maggi R.G., Krämer F. (2019). A review on the occurrence of companion vector-borne diseases in pet animals in Latin America. Parasit. Vectors.

[305] Penzhorn B.L., Vorster I., Redecker G., Oosthuizen M.C. (2016). Confirmation of occurrence of *Babesia vogeli* in a dog in Windhoek, central Namibia. J. S. Afr. Vet. Assoc..

[306] Sibanda D.R. (2011). Molecular Characterization of Tick-Borne Pathogens of Domestic Dogs from Communal Areas in Botswana Hunting Dogs Infected with Multiple Blood-Borne Pathogens. Master’s Thesis.

[307] Dantas-Torres F. (2010). Biology and ecology of the brown dog tick, *Rhipicephalus sanguineus*. Parasit. Vectors.

[308] Walker A.R., Bouattour A., Camicas J.-L., Estrada-Peña A., Horak I.G., Latif A.A., Pegram R.G., Preston P.M. (2003). Ticks of Domestic Animals in Africa: A Guide to Identification of Species.

[309] Dear J.D., Birkenheuer A. (2022). Babesia in North America: An Update. Vet. Clin. N. Am. Small Anim. Pract..

[310] Maggi R.G., Birkenheuer A.J., Hegarty B.C., Bradley J.M., Levy M.G., Breitschwerdt E.B. (2014). Comparison of serological and molecular panels for diagnosis of vector-borne diseases in dogs. Parasit. Vectors.

[311] Shock B.C., Moncayo A., Cohen S., Mitchell E.A., Williamson P.C., Lopez G., Garrison L.E., Yabsley M.J. (2014). Diversity of piroplasms detected in blood-fed and questing ticks from several states in the United States. Ticks Tick Borne Dis..

[312] Bhosale C.R., Wilson K.N., Ledger K.J., White Z.S., Dorleans R., De Jesus C.E., Wisely S.M. (2023). Ticks and Tick-Borne Pathogens in Recreational Greenspaces in North Central Florida, USA. Microorganisms.

[313] Noden B.H., Roselli M.A., Loss S.R. (2022). Effect of Urbanization on Presence, Abundance, and Coinfection of Bacteria and Protozoa in Ticks in the US Great Plains. J. Med. Entomol..

[314] Raghavan R.K., Peterson A.T., Cobos M.E., Ganta R., Foley D. (2019). Current and Future Distribution of the Lone Star Tick, *Amblyomma americanum* (L.) (Acari: Ixodidae) in North America. PLoS ONE.

[315] Modarelli J.J., Westrich B.J., Milholland M., Tietjen M., Castro-Arellano I., Medina R.F., Esteve-Gasent M.D. (2020). Prevalence of protozoan parasites in small and medium mammals in Texas, USA. Int. J. Parasitol. Parasites Wildl..

[316] Shock B.C., Lockhart J.M., Birkenheuer A.J., Yabsley M.J. (2013). Detection of a *Babesia* Species in a Bobcat from Georgia. Southeast. Nat..

[317] Dear J.D., Owens S.D., Lindsay L.L., Biondo A.W., Chomel B.B., Marcondes M., Sykes J.E. (2018). *Babesia conradae* infection in coyote hunting dogs infected with multiple blood-borne pathogens. J. Vet. Intern. Med..

[318] Stayton E., Lineberry M., Thomas J., Bass T., Allen K., Chandrashekar R., Yost G., Reichard M., Miller C. (2021). Emergence of *Babesia conradae* infection in coyote-hunting Greyhounds in Oklahoma, USA. Parasit. Vectors.

[319] Matsuu A., Kawabe A., Koshida Y., Ikadai H., Okano S., Higuchi S. (2004). Incidence of canine *Babesia gibsoni* infection and subclinical infection among Tosa dogs in Aomori Prefecture, Japan. J. Vet. Med. Sci..

[320] Jefferies R., Ryan U.M., Jardine J., Broughton D.K., Robertson I.D., Irwin P.J. (2007). Blood, Bull Terriers and Babesiosis: Further evidence for direct transmission of *Babesia gibsoni* in dogs. Aust. Vet. J..

[321] Irwin P.J. (2009). Canine babesiosis: From molecular taxonomy to control. Parasit. Vectors.

[322] Imre M., Farkas R., Ilie M., Imre K., Hotea I., Morariu S., Morar D., Dărăbuş G. (2013). Seroprevalence of *Babesia canis* Infection in Clinically Healthy Dogs from Western Romania. J. Parasitol..

[323] Joachim A., Unterköfler M.S., Strobl A., Bakran-Lebl K., Fuehrer H.P., Leschnik M. (2023). Canine babesiosis in Austria in the 21st century—A review of cases. Vet. Parasitol. Reg. Stud. Rep..

[324] Castro V.V., da Cruz Boa Sorte Ayres E., Canei D.H., Pereira M.E., Sousa V.R.F., Chitarra C.S., Dutra V., Nakazato L., de Almeida A.B.P.F. (2020). Molecular prevalence and factors associated with *Babesia vogeli* infection in dogs in the Cerrado Mato-Grossense region of Brazil. Cienc. Rural.

[325] Jongejan F., de Vos C., Fourie J.J., Beugnet F. (2015). A novel combination of fipronil and permethrin (Frontline Tri-Act^®^/Frontect^®^) reduces risk of transmission of *Babesia canis* by *Dermacentor reticulatus* and of *Ehrlichia canis* by *Rhipicephalus sanguineus* ticks to dogs. Parasit. Vectors.

[326] Jongejan F., Fourie J.J., Chester S.T., Manavella C., Mallouk Y., Pollmeier M.G., Baggott D. (2011). The prevention of transmission of *Babesia canis canis* by *Dermacentor reticulatus* ticks to dogs using a novel combination of fipronil, amitraz and (S)-methoprene. Vet. Parasitol..

[327] Geurden T., Six R., Becskei C., Maeder S., Lloyd A., Mahabir S., Fourie J., Liebenberg J. (2017). Evaluation of the efficacy of sarolaner (Simparica^®^) in the prevention of babesiosis in dogs. Parasit. Vectors.

[328] Taenzler J., Liebenberg J., Roepke R.K., Heckeroth A.R. (2016). Prevention of transmission of *Babesia canis* by *Dermacentor reticulatus* ticks to dogs after topical administration of fluralaner spot-on solution. Parasit. Vectors.

[329] Beugnet F., Lebon W., de Vos C. (2019). Prevention of the transmission of *Babesia rossi* by *Haemaphysalis elliptica* in dogs treated with Nexgard^®^. Parasite.

[330] Freeman M.J., Kirby B.M., Panciera D.L., Henik R.A., Rosin E., Sullivan L.J. (1994). Hypotensive shock syndrome associated with acute *Babesia canis* infection in a dog. J. Am. Vet. Med. Assoc..

[331] Stegeman J.R., Birkenheuer A.J., Kruger J.M., Breitschwerdt E.B. (2003). Transfusion-associated *Babesia gibsoni* infection in a dog. J. Am. Vet. Med. Assoc..

[332] Wardrop K.J., Birkenheuer A., Blais M.C., Callan M.B., Kohn B., Lappin M.R., Sykes J. (2016). Update on Canine and Feline Blood Donor Screening for Blood-Borne Pathogens. J. Vet. Intern. Med..

[333] Nury C., Blais M.C., Arsenault J. (2021). Risk of transmittable blood-borne pathogens in blood units from blood donor dogs in Canada. J. Vet. Intern. Med..

